# Cellular innovations and diversity in the lepidopteran compound eye

**DOI:** 10.1007/s00359-025-01751-8

**Published:** 2025-07-29

**Authors:** Wei Lu, Marcus R. Kronforst

**Affiliations:** Department of Ecology and Evolution, University of Chicago, Chicago, IL 60637 USA

**Keywords:** Butterfly, Color vision, Filtering pigment, Moth, Ommatidia, Opsin, Photoreceptor, Spectral sensitivity

## Abstract

Lepidoptera, having co-diversified with flowering plants and adapted to various diel niches, present a remarkable system for studying compound eye cell type diversity. Here we synthesize the latest research regarding Lepidopteran eye evolution across different timescales, from species-level variation to family-level changes, and mechanistic levels, from broad anatomical variation to molecular mechanisms responsible for spectral tuning. Opsin duplication, differential expression, and co-expression, combined with lateral filtering pigments, generate diverse spectral sensitivities in photoreceptors. Lateral filtering is particularly important for the convergent evolution of red vision. These diverse photoreceptors combine to form a handful of ommatidial types distributed differentially across eye regions, potentially specializing for distinct behavioral tasks. The coordinated development of these complex retinal mosaics requires precise regulatory mechanisms that we are only beginning to understand. Notably, only a subset of these ommatidial types contribute to color vision, highlighting the need for more research on their roles in motion and polarization vision. We also review support cells providing essential functions such as light insulation or reflection. Future research should focus on identifying ecological pressures driving visual system evolution, genetic bases of diverse retinal mosaics, and neural integration of visual information in Lepidoptera.

**Supplementary Information:**

The online version contains supplementary material available at 10.1007/s00359-025-01751-8.

## Introduction

Typical of crustaceans and hexapods (including insects), the compound eye is one of two principal visual systems in the animal kingdom, alongside the single-lens camera-type eyes found in vertebrates and cephalopods (Harzsch and Hafner 2006). Butterflies and moths (Lepidoptera) represent one of four major insect superradiations, alongside Coleoptera, Hymenoptera, and Diptera, with most lineages diversifying rapidly with the rise of flowering plants (angiosperms) in the Cretaceous (Heikkilä et al. 2012; Wahlberg et al. 2013; Mitter et al. 2017; Espeland et al. 2018; Chazot et al. 2019; Kawahara et al. 2019). As a predominantly herbivorous clade, Lepidoptera has among the fastest diversification rates of any insect order (Ehrlich and Raven 1964; Wiens et al. 2015; Kawahara et al. 2023). This close association with angiosperms, as herbivores during the larval stage and pollinators as adults, has likely driven the remarkable diversification of the lepidopteran visual system in order to identify suitable host plants and detect flowers. Additionally, Lepidoptera exhibit a wide range of diel activity patterns (day vs. night), with more than 40 independent transitions to diurnality, further driving the diversification of the Lepidoptera compound eye (Kawahara et al. 2018).

Numerous comprehensive reviews have explored insect color vision and the diversity of retinal mosaics (Briscoe and Chittka 2001; Stavenga and Arikawa 2006; Wernet et al. 2015; Arikawa 2017; Song and Lee 2018; Schnaitmann et al. 2020; van der Kooi et al. 2021; McCulloch et al. 2022a). However, recent developments have created new opportunities to expand on this foundation. The availability of high-quality Lepidoptera genomes has greatly enhanced our ability to investigate the genetic basis of visual diversity (Mulhair et al. 2023; Wright et al. 2024a). Furthermore, an increasing number of studies have linked compound eye structure and function to butterfly behavior, ecology, and evolution (Wainwright et al. 2023; Rossi et al. 2024; Wright et al. 2024b; Dang et al. 2025; VanKuren et al. 2025). Together, these advances underscore the need for a comprehensive, up-to-date review of the lepidopteran visual system. In this review, we summarize both shared patterns and clade-specific features of compound eye cell types in the Lepidoptera.

## Basic structure of the Lepidoptera compound eye

**Fig. 1:**
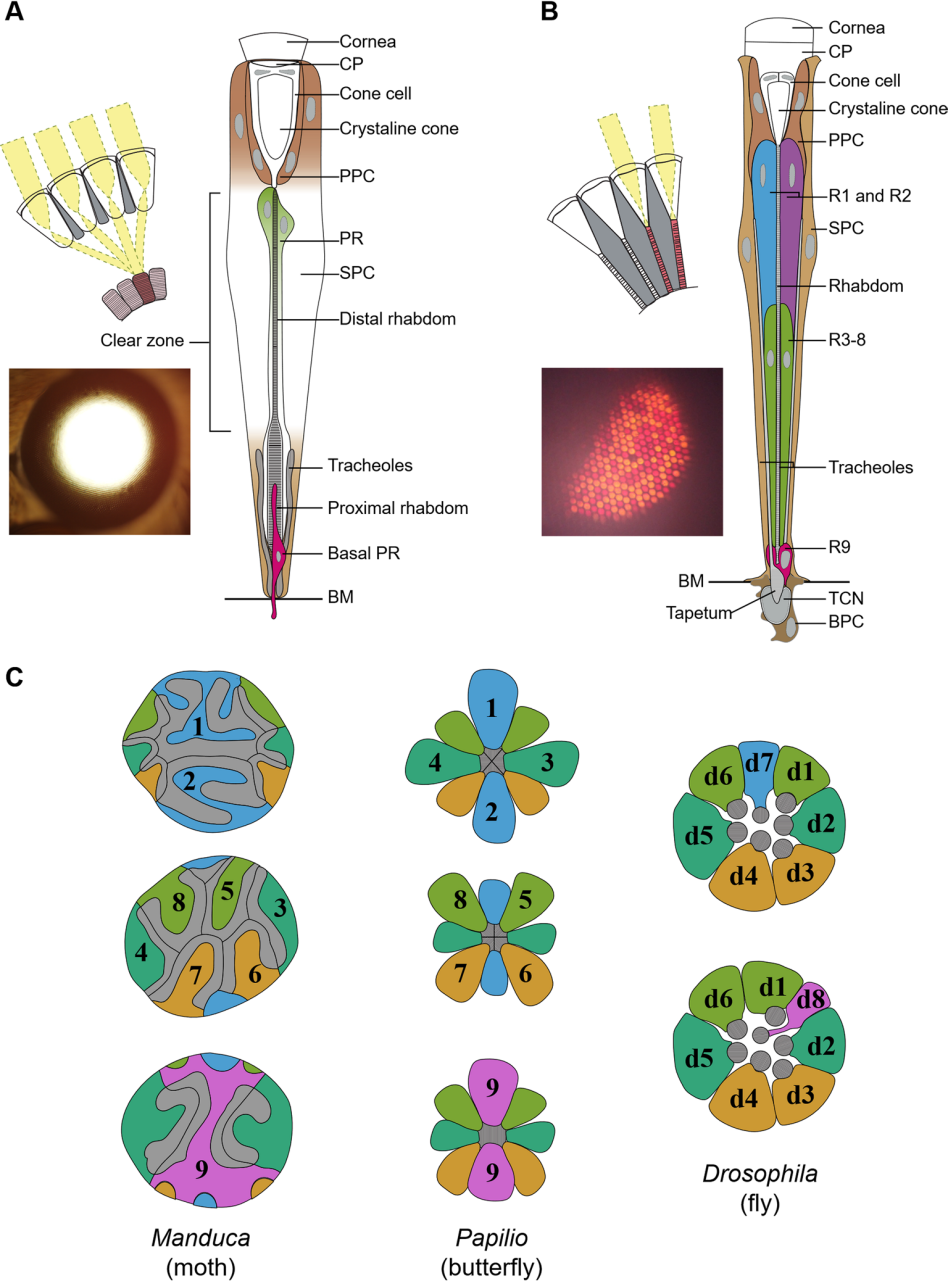
Anatomy and light paths of superposition and apposition compound eyes. **A** *Right side*: Anatomy of a superposition eye in the nocturnal corn borer moth (*Ostrinia nubilalis*), adapted from (Belušič et al. 2017). The dioptric apparatus (cornea and crystalline cone) in the distal region is separated from the proximal rhabdom by a clear zone. The nuclei of the basal PRs lie directly beneath the rhabdom. *Top left side*: Light path in a superposition eye, where light from multiple ommatidia passes through the clear zone and converges on a single proximal rhabdom, enhancing light sensitivity at the expense of acuity. *Lower left side*: Superposition eyeshine image of *Helicoverpa armigera*. Image courtesy of Dr. Kentaro Arikawa. **B** *Right side*: Anatomy of an apposition eye in the diurnal small tortoiseshell butterfly (*Aglais urticae*), adapted from (Kolb 1985). The bilobed basal PR (R9) has its nucleus positioned adjacent to the rhabdom. *Top left side*: Light path in an apposition eye, where each ommatidium is optically isolated by heavily pigmented SPCs; only light entering at specific angles reaches the rhabdom. *Lower left side*: Apposition eyeshine image of *Heliconius cydno*. **C** Cross sections of the rhabdoms in different tiers. The top row is the distal tier, and the bottom row is the most proximal tier. Homology relationships among *Manduca* (modified from White et al. 2003), *Papilio* (modified from Arikawa and Stavenga 1997), and *Drosophila* (modified from Reinke and Zipursky 1988) are indicated by the matching colors. PR, photoreceptor; BM, basement membrane; PPC, primary pigment cell; SPC, secondary pigment cell; BPC, basal pigment cell; CP, corneal process

The compound eye consists of many repeated individual units called ommatidia. Most butterflies (superfamily Papilionoidea) studied thus far have the ancestral afocal apposition eye (Fig. 1B), where the lens in each ommatidium forms a small, inverted image (Exner 1891; Nilsson 1989; Land and Nilsson 2012; Meyer-Rochow and Lindström 2025). Another major compound eye type, the refracting superposition eye (Fig. 1A), is found in diurnal Hesperiidae (Orridge et al. 1972), nocturnal Hedylidae (Yack et al. 2007), and many moth families (Pirih et al. 2018). Unlike apposition eyes, superposition eyes form a single erected image deeper in the eye by combining light from many lenses (Exner 1891; Nilsson 1989; Land and Nilsson 2012; Meyer-Rochow and Lindström 2025). A pigment-free clear zone exists between the dioptric structures and the proximal light-sensing receptors, allowing light entering from different lenses to pass through. An intermediate eye type, which lacks the clear zone but otherwise resembles a superposition eye, has also been found in several miniature moth species, likely reflecting the theoretical size limit imposed by superposition optics. (Meyer-Rochow and Gál 2004; Honkanen and Meyer-Rochow 2009; Fischer et al. 2012, 2014).

The differences between superposition and apposition eyes can also be distinguished by their eyeshine. Light entering the ommatidia reaches a reflective structure formed by tracheae, known as the tapetum, which reflects the light back and produces the eyeshine. Dark-adapted moths with superposition eyes exhibit a circular glow visible to the naked eye when illuminated (Fig. 1A). In contrast, many diurnal butterflies with apposition eyes display colorful and sometimes heterogeneous eyeshine (Fig. 1B), due to the reflecting tapetum at the base of each ommatidium (Exner 1891; Miller and Bernard 1968). The eyeshine represents the light not absorbed by the pigments within each ommatidium (Stavenga 2002).

Each ommatidium contains photoreceptors as well as support cells such as pigment and cone cells. Photoreceptors are sensory neurons that detect light and convert it into electrical signals. The canonical insect ommatidium contains eight photoreceptors, which can be developmentally subdivided based on their anatomical positions, specification sequences, and axonal projections. In *Drosophila*, photoreceptors are classified as outer or inner photoreceptors according to the position of their rhabdomeres within the open rhabdom (Friedrich et al. 2011). The rhabdomeres of the two inner photoreceptors are stacked in tandem and located centrally within the interrhabdomeral space. These inner photoreceptors are referred to as dR7 (distal) and dR8 (proximal), where the ‘d-’ prefix denotes the naming scheme by Dietrich (1909) in his study of the retinal organization in higher Diptera. The inner photoreceptors are long visual fibers (LVFs), projecting to the medulla, whereas the outer photoreceptors are short visual fibers (SVFs), projecting to the lamina. In butterflies, a different photoreceptor naming scheme was introduced by Ribi (1978) in his description of the retinal structure of *Pieris rapae*. Ribi (1978) named photoreceptors R1-9 based on the position of their nuclei along the rhabdom and their orientation. Although butterflies have a fused rhabdom without interrhabdomeral space, photoreceptors R1, R2, and R9 were initially identified as homologous to inner photoreceptors in *Drosophila* due to their projections to the medulla (Ribi 1987; Shimohigashi and Tominaga 1991, 1999). Specifically, R1 and R2, which have distal rhabdomeres, correspond to dR7, while the basal R9 corresponds to dR8 (Friedrich et al. 2011). These homology assignments (Fig. 1C) are further supported by similarities in cell body positioning and the sequence of photoreceptor specification (Gao et al. 2025). Notably, R9 is unique in that both its cell body and rhabdomere are highly restricted to the most proximal position. Furthermore, a recent study suggests that in *Papilio*, R9 cells are SVFs terminating in the lamina rather than the medulla, in contrast to dR8 in *Drosophila* (Matsushita et al. 2022).

**Fig. 2:**
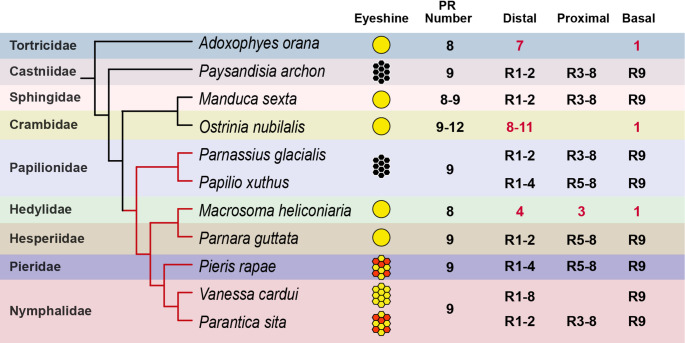
Evolution of ommatidial anatomical structures in Lepidoptera. A phylogeny of representative species from various Lepidoptera families is shown, with butterflies (Superfamily Papilionoidea) highlighted in red branches. The family-level phylogeny is based on (Kawahara et al. 2019). In the eyeshine column: colorful hexagons, apposition eyes with heterogeneous eyeshine; yellow hexagons, apposition eyes with homogeneous eyeshine; black hexagons, apposition eyes without eyeshine; yellow circles, superposition eyes with eyeshine. For each species, the total number of photoreceptors per ommatidium is indicated, along with their grouping based on their contribution to the rhabdom, which is organized into two or three tiers. Photoreceptor naming follows the Ribi (1978) scheme. For species where photoreceptor homologies are uncertain, the number of photoreceptors in each tier is indicated (in red text). Across all species, regardless of eye type (apposition or superposition), the ommatidium consistently contains a distinct basal photoreceptor. References: *Adoxophyes* (Satoh et al. 2017); *Paysandisia* (Pirih et al. 2018); *Manduca* (White et al. 2003; Gao et al. 2025); *Ostrinia* (Belušič et al. 2017); *Parnassius* (Matsushita et al. 2012); *Papilio* (Arikawa and Uchiyama 1996); *Macrosoma* (Yack et al. 2007); *Parnara* (Shimohigashi and Tominaga 1986); *Pieris* (Ribi 1978); *Vanessa* (Briscoe et al. 2003); *Parantica* (Nagloo et al. 2020)

In contrast to the well-studied higher Diptera ommatidium, which contains one dR7 and one dR8 inner photoreceptor, the butterfly ommatidium includes an additional inner photoreceptor (two dR7 and one dR8). Among the major winged insect (Pterygota) orders, this configuration of two dR7 cells has only been observed in Lepidoptera and Hymenoptera, two groups that have been studied extensively in the context of color vision (van der Kooi et al. 2021; Gao et al. 2025). The nocturnal moth-butterfly (Hedylidae) represents a notable outlier within the butterfly superfamily, possessing only eight photoreceptors per ommatidium (Yack et al. 2007). In contrast, outside the butterfly superfamily, the number of photoreceptors within ommatidia is more variable (Fig. 2). For example, the hawkmoth *Manduca sexta* has fly-like ommatidia (one dR7 homolog) in the dorsal region and butterfly-like ommatidia (two dR7 homologs) in the ventral region (White et al. 2003; Gao et al. 2025). In moth species with superposition eyes, each ommatidium can contain 8–16 photoreceptors (Horridge and Giddings 1971; Horridge et al. 1977; Meyer-Rochow and Lau 2008; Belušič et al. 2017; Yang et al. 2024).

The key structural feature of photoreceptors is the rhabdomere, a dense array of microscopic membrane protrusions known as microvilli, where a high density of visual pigments is found within the microvillar membrane (Osorio 2007). Microvilli absorb plane-polarized light most efficiently when their orientation is parallel to the light’s e-vector (Labhart and Meyer 2002). The rhabdomeres of all photoreceptors within an ommatidium collectively form the rhabdom. In Lepidoptera, these rhabdomeres are closely packed together into a single fused rhabdom. The combination of fused rhabdom and apposition eye is thought to represent the arthropod ancestral state (Osorio 2007). In butterflies with apposition eyes, the rhabdoms are typically thin and rod-shaped, whereas in moths with superposition eyes, they often exhibit star-like or rosette-shaped configurations (Meyer-Rochow and Lindström 2025).

The spatial arrangement of the rhabdom is quite variable (Fig. 2). For instance, in Papilionidae and Pieridae, the rhabdom is fully tiered: R1-4 cells contribute microvilli to the distal tier of the rhabdom, while the proximal tier consists of R5-8 microvilli. At the most basal position, R9 contributes to a small section of the rhabdom (Ribi 1978; Arikawa and Uchiyama 1996). In contrast, species in the family Nymphalidae generally have incompletely tiered rhabdoms, where R3-8 contribute their microvilli along the entire length of the rhabdom (Gordon 1977). Exceptions to these patterns occur in some butterfly and moth species. For example, the giant butterfly-moth (*Paysandisia archon*) has two types of ommatidia. In type I, the distal rhabdom consists exclusively of R1/2, and this configuration is also found in the butterfly *Parnassius glacialis* (Matsushita et al. 2012). In type II, the distal rhabdom is split into two sub-rhabdoms, one formed by R2, R3, R5, R6 and the other by R1, R4, R7, R8 (Pirih et al. 2018).

## Evolution of lepidopteran opsin genes

**Fig. 3:**
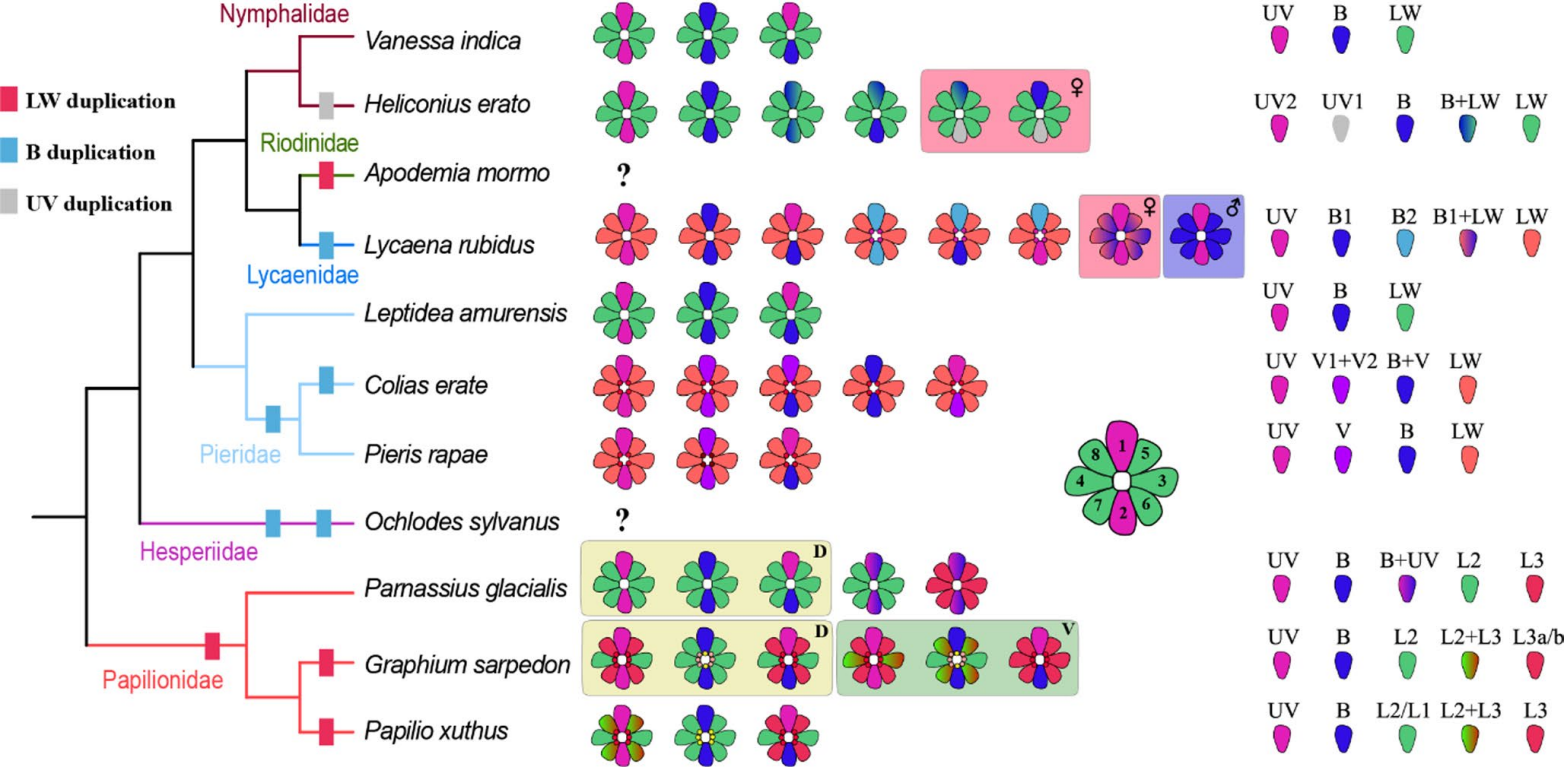
Evolution of retinal mosaics in butterflies. *Left panel*: the phylogeny of several butterfly species with well-characterized opsin expression patterns, with gene duplication events marked along the branches. The phylogeny is based on (Heikkilä et al. 2012; Espeland et al. 2018; Kawahara et al. 2019, 2023). *Middle panel*: ommatidial types for each species based on opsin expression. The enlarged ommatidium indicates the position of R1–R8 cells (R9 not shown). Types that are sex-specific or region-specific are highlighted with boxes (D: dorsal, V: ventral). The presence of perirhabdomal filtering pigments is indicated for *Colias*, *Pieris*, *Graphium*, and *Papilio*. *Right panel*: the diversity of photoreceptor types that compose the retinal mosaics. Co-expression is indicated by mixed colors within a cell and plus signs. References: *Vanessa* (Briscoe et al. 2003; Pirih et al. 2020); *Heliconius* (McCulloch et al. 2017); *Apodemia* (Frentiu et al. 2007); *Lycaena* (Sison-Mangus et al. 2006); *Leptidea* (Uchiyama et al. 2013); *Colias* (Ogawa et al. 2012); *Pieris* (Arikawa et al. 2005); *Ochodes* (Mulhair et al. 2023); *Parnassius* (Awata et al. 2010); *Graphium* (Chen et al. 2016); *Papilio* (Arikawa 2003)

The spectral sensitivity of photoreceptors is primarily determined by the visual pigments they express. In arthropods, these visual pigments are composed of rhabdomeric-type opsin (r-opsin) proteins, members of the G protein-coupled receptor family, that covalently bind to the retinal-based chromophores and respond to different wavelengths of light (Henze and Oakley 2015). Ancestrally, Lepidoptera possess three types of r-opsins with distinct peak sensitivity: green-sensitive long-wavelength (LW) opsins, blue-sensitive short-wavelength (B) opsins, and ultraviolet-sensitive (UV) opsins (Briscoe and Chittka 2001; Stavenga and Arikawa 2006; Briscoe 2008). Most photoreceptors follow the ‘One Receptor’ rule of sensory neurons, expressing a single opsin gene per cell (Mazzoni et al. 2004). However, numerous instances of opsin co-expression have been observed in butterfly photoreceptors (Fig. 3).

Outer photoreceptors (R3-8) mainly express LW opsins. The inner photoreceptor R9 has also been shown to express LW opsins in species such as *Papilio glaucus* and *Vanessa cardui* (Briscoe et al. 2003; Briscoe 2008). However, due to its small size and basal position within the ommatidium, the opsin expression of R9 remains poorly understood in most species. Inner photoreceptors R1 and R2 typically express UV or B opsins. Stochastic expression of UV or B opsins in R1/2 results in three stochastically distributed ommatidial types: UV-UV, UV-B, and B-B (Perry et al. 2016). This type of retinal mosaic is typical of most butterflies and moths (White et al. 2003; Arikawa 2003) and is also found in honeybees (Wakakuwa et al. 2005).

One key mechanism for expanding the spectral diversity of photoreceptors is gene duplication and divergence. Duplicated opsins can evolve distinct peak sensitivities by changing amino acids in the chromophore-binding pocket, also known as spectral tuning. These opsin paralogs can acquire novel expression patterns in new cell types or specialize among subsets of the original cell type (Briscoe 2008). Gene duplications of opsins in Lepidoptera have been documented since the early-day cDNA cloning and in situ hybridization studies (Kitamoto et al. 1998; Briscoe 2000). Following the publication of the first moth genome (*Bombyx mori*; Xia et al. 2004) and the first butterfly genome (*Danaus plexippus*; Zhan et al. 2011), an increasing number of lepidopteran genomes and transcriptomes have been sequenced using next-generation sequencing technologies. These datasets have enabled broader taxonomic surveys of opsin gene diversity (Sondhi et al. 2021; Kuwalekar et al. 2022). However, opsin gene copy number may be underestimated in fragmented genome assemblies. This limitation is now being addressed with chromosome-level genome assemblies produced using third-generation sequencing methods, such as those generated by the Darwin Tree of Life project (Mulhair et al. 2023). Opsin gene duplications are now recognized as more widespread across Lepidoptera than previously thought (Table S1).

### Long-wavelength opsin duplication and expression

LW opsin duplications are widespread across Lepidoptera (Sondhi et al. 2021; Kuwalekar et al. 2022; Mulhair et al. 2023). Within the butterfly superfamily, LW opsin duplications have been identified in Papilionidae, Riodinidae, Nymphalidae, and Hesperiidae (Fig. 3). They are also common across multiple moth families. One of the most ancient opsin duplication events in Lepidoptera is the duplication of LW opsin in the Noctuoidea superfamily, which occurred approximately 80 million years ago. All current Noctuoidea species share an intronless *LWS2* gene, likely produced by the retrotransposition of the ancestral *LWS1* copy (Mulhair et al. 2023).

At the base of Papilionidae, an LW opsin duplication event generated two opsins: the ancestrally green-sensitive L2 and the red-sensitive L3. The peak absorption wavelength of L3 is approximately 570 nm in *Papilio xuthus* (Kitamoto et al. 1998; Saito et al. 2019). *Parnassius glacialis* butterflies (subfamily Parnassiinae) only have L2 and L3 LW opsin copies from the ancestral duplication (Awata et al. 2010). In contrast, *Graphium sarpedon* (subfamily Papilioninae), a butterfly with extreme spectral richness, possesses three LW opsins (L2, L3a, and L3b), due to a duplication of L3 that is shared among the Leptocircini tribe. In *Graphium*, dorsal R3-8 photoreceptors only express one LW opsin per cell (either L2 or L3a), while ventral R3-8 photoreceptors can co-express two or three LW opsins in a single photoreceptor, generating at least five types of long-wavelength-sensitive photoreceptors (Chen et al. 2016). In *Papilio* butterflies, a separate, genus-specific duplication of L3 produced three total LW opsins: L2, L3, and L1. Similar to *Graphium*, each R3-8 photoreceptor can express one or two LW opsins, although the co-expression of all three LW opsins has not been observed in *Papilio* (Kitamoto et al. 1998; Briscoe 2008).

Although LW opsin duplications have been documented in both diurnal and nocturnal Lepidoptera, not all duplicated copies function in color vision or brightness contrast. For example, in *Bombyx mori*, one duplicated LW opsin is expressed in the larval brain tissue, where it regulates photoperiodic responses (Shimizu et al. 2001).

### Blue opsin duplication and expression

The most well-characterized B opsin duplication events have been documented in the butterfly families Lycaenidae and Pieridae (Fig. 3). In Lycaenidae, an ancestral B opsin duplication gave rise to two B opsin copies, B1 and B2, which are shared across the family (Bernard and Remington 1991; Sison-Mangus et al. 2006). These opsins are expressed in R1/2 photoreceptors in distinct, non-overlapping patterns with each other and with UV opsins. As a result, *Lycaena rubidus* exhibits six R1 and R2 subtype combinations: UV-UV, UV-B1, UV-B2, B1-B1, B1-B2, and B2-B2 (Sison-Mangus et al. 2006). In *L. rubidus*, B1 also shows a novel expression pattern in R3-8 photoreceptors, which ancestrally expressed only LW opsins. In females, these photoreceptors in the dorsal eye co-express LW and B1 opsins, while in males, the same cells only express B1. This sexually dimorphic expression pattern has been linked to sexual selection and the prevalence of blue pigments on Lycaenidae wings (Sison-Mangus et al. 2006).

Two blue opsin duplication events have been identified in Pieridae. The first is an ancestral blue opsin duplication that occurred at the base of the Coliadinae and Pierinae lineages, generating a blue-sensitive opsin (B, λ_max_ at 450 nm) and a violet-sensitive opsin (V, λ_max_ at 420 nm) with a spectral shift toward the UV range (Wakakuwa et al. 2010). In Coliadinae, the V opsin underwent a second duplication (Arikawa et al. 2005; Awata et al. 2009). As a result, Coliadinae species possess three blue opsins (B, V1, V2), while Pierinae species have two (B and V). Surprisingly, the duplication of blue opsins does not increase the total number of ommatidial types in *Pieris rapae*; only three ommatidial types (UV-UV, UV-B, and V-V) are observed. Notably, V opsins are expressed only in ommatidia that lack the ancestral UV or B opsin expression (Arikawa et al. 2005).

In *Colias erate*, the violet opsins V1 and V2 are always co-expressed in R1/2 photoreceptors. Additionally, a novel photoreceptor subtype has been identified in *Colias* that expresses all three blue opsins (B, V1, and V2), representing the highest number of co-expressed opsin genes within a single photoreceptor (Ogawa et al. 2012). Beyond the well-characterized blue opsin duplications in Pieridae and Lycaenidae, similar duplications have also been reported in several Hesperiidae butterflies and even in an Erebidae moth species (Mulhair et al. 2023).

### Ultraviolet opsin duplication and expression

Unlike LW and B opsin duplications, UV opsin duplications are rare in Lepidoptera. The only confirmed UV opsin duplication event occurred in the common ancestor of all *Heliconius* butterflies, generating UV1 and UV2 (Briscoe et al. 2010). In the *erato*/*sara*/*sapho* clade, the ancestral *UVRh2* is located on an autosome but the duplicated *UVRh1* is located on the female-specific W chromosome, resulting in sexually dimorphic UV opsin expression (Chakraborty et al. 2023). In females of this clade, two distinct UV photoreceptor cell types have been identified, each expressing either UV1 or UV2 (McCulloch et al. 2016, 2017). Behavioral studies further support this sexual dimorphism, showing that female *H. erato* and *H. charithonia* possess true UV color vision (Finkbeiner and Briscoe 2021; Chakraborty et al. 2023).

In the other major *Heliconius* clade (*melpomene*/*doris*), both *UVRh1* and *UVRh2* are located on autosomes. Since the sister group of this *melpomene*/*doris* clade, *H. aoede*, only expresses *UVRh2* in males (based on RNA-seq data), the most parsimonious explanation is that *UVRh1* was initially duplicated onto the W chromosome and later translocated to an autosome in the *melpomene*/*doris* lineage (McCulloch et al. 2017). Within this clade, female *H. doris* have an additional UV photoreceptor cell type that co-expresses UV1 and UV2 almost equally, while *H. ethilla* in the silvaniform lineage lost UV2 expression entirely due to the pseudogenization (McCulloch et al. 2017). Even within a single *H. cydno* species complex, peak sensitivities of UV photoreceptors vary significantly across subspecies and sexes, which are driven by shifts in the relative expression level of UV1 and UV2 (Buerkle et al. 2022; VanKuren et al. 2025).

Overall, a single genus-specific UV opsin duplication event, followed by chromosomal translocation and lineage-specific pseudogenization, has resulted in at least eight distinct R1/2 ommatidial types (McCulloch et al. 2017). This complex pattern of gene expression evolution highlights that understanding spectral diversity requires not only broad taxonomic sampling, but also dense sampling within genera, as closely-related species can exhibit substantial differences.

### Co-expression of multiple opsins

As noted previously, photoreceptors broaden their spectral sensitivity by co-expressing multiple opsin genes within the same cell (Arikawa et al. 2003). For example, *Colias* butterflies co-express V1 and V2 opsins, derived from a duplication at the base of the Coliadinae subfamily (Ogawa et al. 2012). Similar co-expression of opsins originating from genus- or family-level duplications is also observed in other species (Arikawa et al. 2003; Briscoe et al. 2010; Chen et al. 2016).

In contrast, the co-expression of opsins from different spectral classes (UV, B, LW) is much rarer in Lepidoptera. In *Parnassius glacialis*, a subset of ventral R1/2 photoreceptors co-express UV and B opsins (Awata et al. 2010), similar to the ventral stripe dR7 photoreceptors of the mosquito *Aedes aegypti* (Hu et al. 2011). Even more surprising is the co-expression of B and LW opsins, which are typically restricted to inner and outer photoreceptors, respectively. In *Lycaena rubidus*, female R3-8 photoreceptors co-express B1 and LW opsins (Sison-Mangus et al. 2006). Across the Heliconiini clade (including *Heliconius*, *Eueides*, and *Dryas*), multiple retinal mosaics feature R1/2 photoreceptors that co-express B and LW opsins (McCulloch et al. 2017). These broad-spectrum photoreceptors generate three additional ommatidial types (McCulloch et al. 2017; Chakraborty et al. 2023). Together, these examples illustrate the remarkable flexibility of opsin expression in Lepidoptera, particularly the unexpected expression of outer photoreceptor opsins in inner photoreceptors, and vice versa.

### Temporal opsin expression pattern

The first clusters of differentiated photoreceptors appear during the wandering larval stage in *Manduca* moths (Monsma and Booker 1996; Champlin and Truman 1998). However, the rhabdom is not completed until the end of pupal development or shortly after adult eclosion (Monsma and Booker 1996; Arikawa et al. 2017). In *Papilio xuthus*, the onset of opsin gene expression occurs during pupal development and follows a consistent temporal sequence: UV and B opsins are expressed first, followed by L2, then L3, and finally L1 (Arikawa et al. 2017). The ancestral green-sensitive L2 opsin initially appears in all R3-8 photoreceptors. In a subset of ommatidia, L2 is later replaced by the red-sensitive L3 in R5-8 photoreceptors. The genus-specific L1 opsin is only detectable after day 9 and is restricted to R3/4 photoreceptors, which continue to co-express L2 (Arikawa et al. 2017). Interestingly, the temporal order of opsin expression in *P. xuthus* mirrors the evolutionary sequence in which these opsins arose, suggesting a case of “ontogeny recapitulating phylogeny”(Domazet-Lošo and Tautz 2010; Kalinka et al. 2010). Whether this pattern holds true for other duplicated opsins remains unknown. More comparative studies on the temporal expression pattern of duplicated opsin genes need to be done, especially in species with multiple opsin duplications, such as the *Colias* butterflies with their three middle-wavelength opsins.

## Lateral filtering and convergent evolution of red photoreceptors

**Fig. 4:**
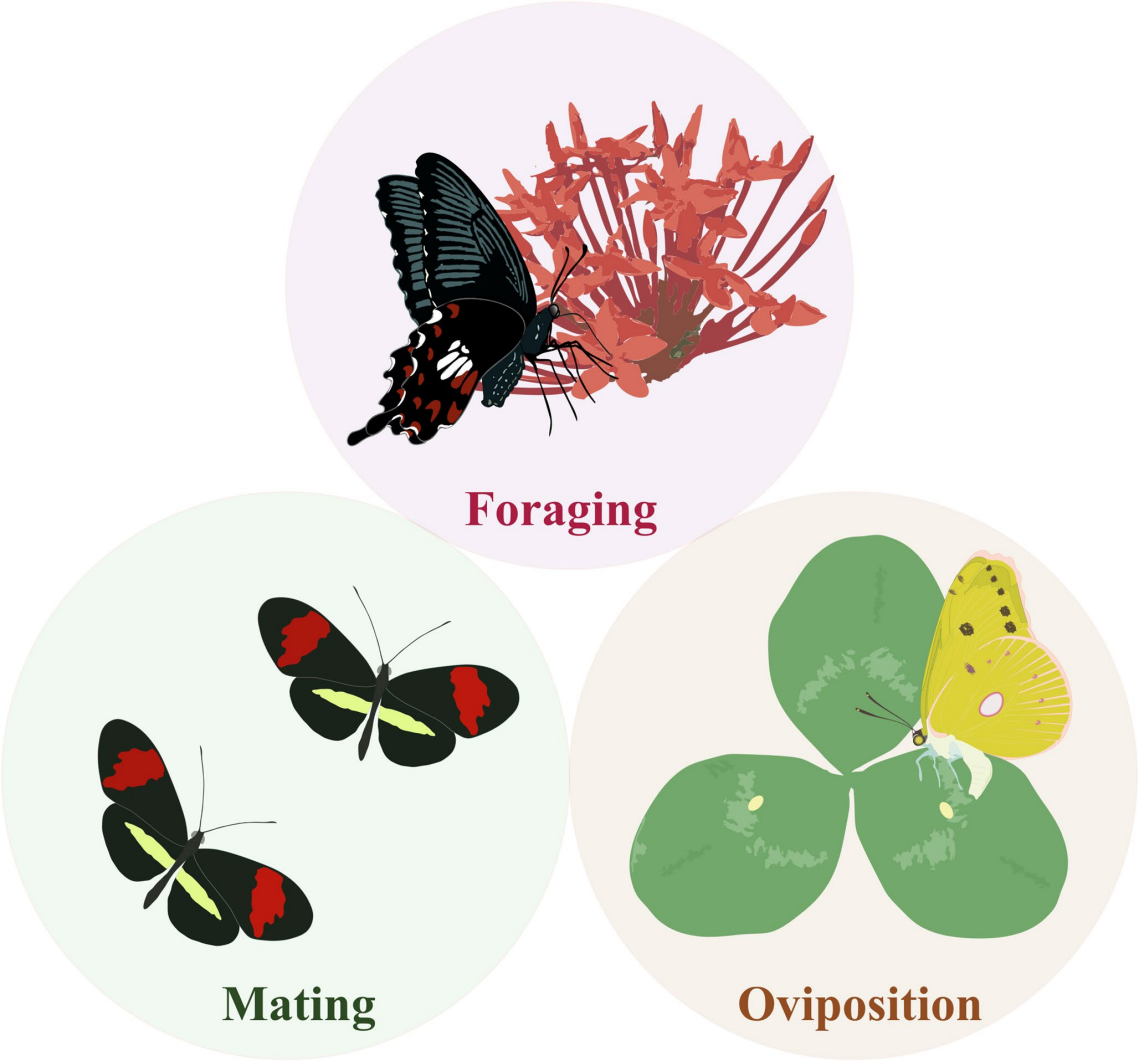
Behavioral ecology of red color vision. *Top*: *Papilio polytes* (family Papilionidae) feeding on red *Ixora* flowers. *Lower left*: A male *Heliconius melpomene* (family Nymphalidae) chasing a conspecific female with bright red patches on the forewings. *Lower right*: *Colias erate* (family Pieridae) laying eggs on *Trifolium* leaves

The evolution of red color vision may serve multiple functions, including mate recognition, flower detection, and host plant discrimination for oviposition (Fig. 4). While red-sensitive photoreceptors (λmax > 565 nm) are rare in Hymenoptera, they have evolved repeatedly and are widespread in Lepidoptera, especially among diurnal butterflies (Briscoe and Chittka 2001). The evolution of red-sensitive photoreceptors both expands the visual range and enhances wavelength discrimination in the long-wavelength spectrum.

Sensitivity to long wavelengths, including red light, allows butterflies to exploit nectar-rich red flowers, which are typically pollinated by birds and avoided by bees (Johnson and Bond 1994; Chen et al. 2020b). Butterflies in the family Papilionidae, Pieridae, and Nymphalidae are common visitors to these butterfly-pollinated flowers (Hirota et al. 2013; Kiepiel and Johnson 2014). In addition to flower discrimination, the evolution of red-sensitive receptors may aid butterflies in selecting young versus mature leaves for oviposition (Kelber 1999).

True nocturnal color vision has been demonstrated in three hawk moth species (Sphingidae), where it might enhance flower detection during foraging (Kelber et al. 2002, 2003; Warrant and Somanathan 2022). It can also play a role in oviposition. In the tomato leafminer moth, *Tuta absoluta*, mutations in either the B or LW opsins have been shown to alter host plant preferences (Tang et al. 2024).Though uncommon, red-sensitive photoreceptors have been identified in multiple nocturnal moth species across diverse families, although the ecological significance of red color vision in these species remains unclear (Langer et al. 1979; Eguchi et al. 1982; Satoh et al. 2016; van der Kooi et al. 2021).

The presence of red photoreceptors can be detected in several ways: behaviorally through color discrimination tests, functionally by electrophysiology, or indirectly by the identification of red filtering pigments via histology or eyeshine. However, despite the widespread occurrence of red photoreceptors in Lepidoptera, previous attempts to link their evolution to behavioral or ecological traits have failed to identify consistent selective pressures across lineages (Briscoe and Chittka 2001).

### Filtering pigments in Lepidoptera compound eyes

The spectral sensitivity of photoreceptors is determined not only by the photosensitive opsins they express but also by the presence of photostable filtering pigments within the ommatidia. These photostable pigments are stored in membrane-bound compartments known as pigment granules, which are lysosome-related organelles (Dell’Angelica et al. 2000). Pigment granules are found in both pigment cells and photoreceptors, most of which exhibit relatively uniform absorption across the 300–700 nm wavelength range (Langer and Struwe 1972). In both butterflies and moths, the migration of these pigment granules within an ommatidium function like a pupil, regulating light input to the rhabdom during light adaptation (Stavenga and Kuiper 1977; Satoh et al. 2017). In superposition eyes, two types of pupil mechanisms are involved in light adaptation. In most nocturnal moths, pigment granules of secondary pigment cells (SPCs) migrate across the clear zone. In diurnal moths and skipper butterflies, pigment granules of primary pigment cells (PPCs) migrate around the proximal tip of the crystalline cone (Warrant and McIntyre 1996). In some small nocturnal moths, both mechanisms are combined during light adaptation (Warrant and McIntyre 1996). Pupillary response in butterflies with apposition eyes also involve the radial migration of pigment granules within photoreceptors, in addition to pigment granule migration in SPCs and contraction of PPCs (Stavenga and Kuiper 1977; Ribi 1978). Pigment granules of pigment cells can also absorb stray light from adjacent ommatidia, ensuring that each ommatidium primarily receives axial light. This function enhances visual acuity in species with apposition-type eyes (Linzen 1974).

**Fig. 5:**
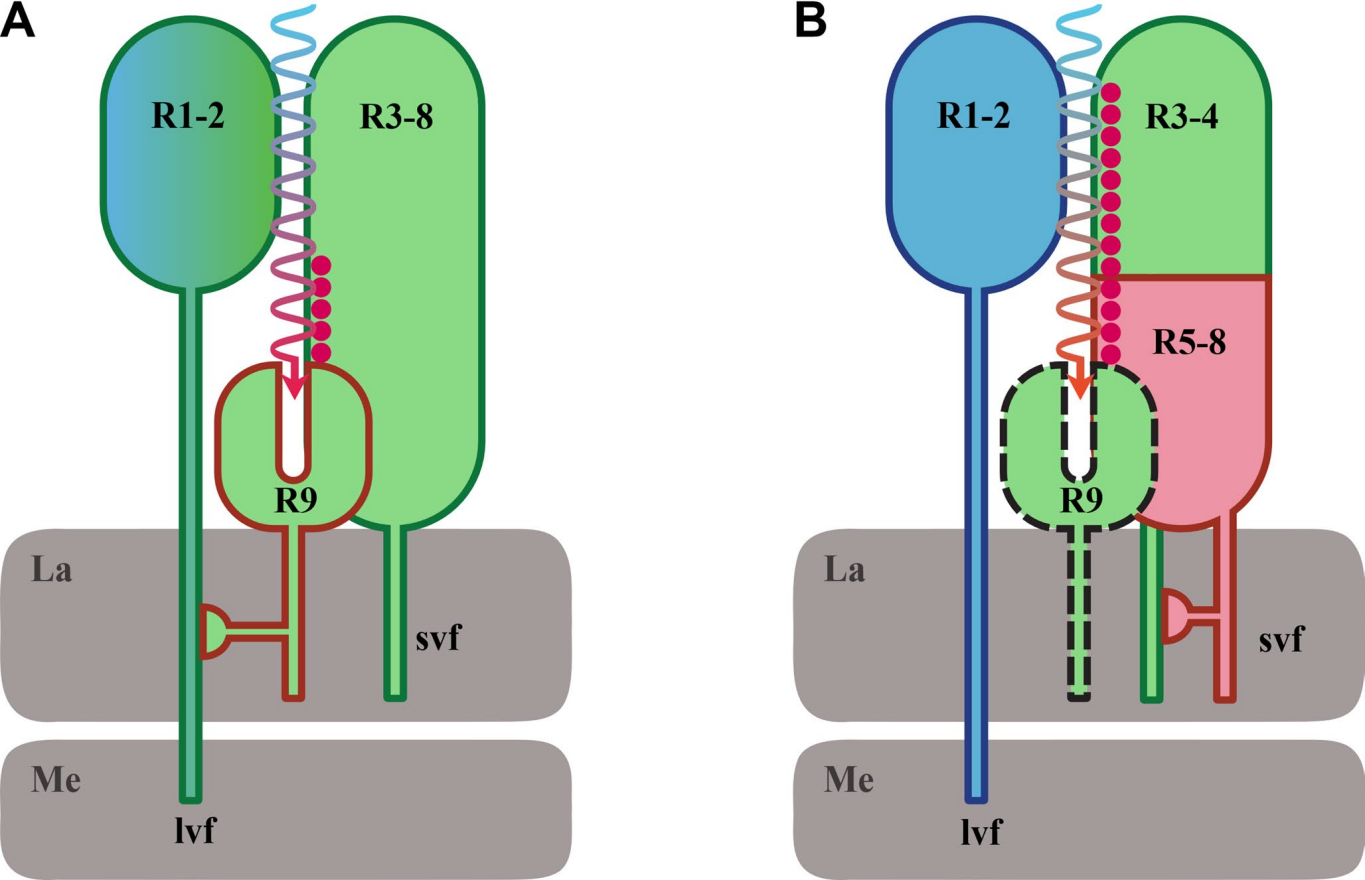
Convergent evolution of red-green opponency. Schematic representation of red-green opponency mechanisms in Nymphalidae (**A**) and Papilionidae (**B**). Photoreceptor outline colors indicate green-, blue-, or red-sensitive cells, while the fill colors represent the opsins they express. **A** In Nymphalidae, green-sensitive R1 or R2 photoreceptors (which co-express B and LW opsins) receive direct inhibitory input from red-sensitive R9 photoreceptors (Belušič et al. 2021). The presence of red perirhabdomal pigments shifts the sensitivity of R9 photoreceptors from green to red. **B** In Papilionidae, the role of R9 remains unclear (marked with a dashed line). Green-sensitive R3–4 receive inhibitory input from proximal red-sensitive R5–8, driven by a combination of red-sensitive opsin expression and lateral filtering (Chen et al. 2020a). La, lamina; Me, medulla; lvf, long visual fiber; svf, short visual fiber

Beyond functioning as pupil filters, some pigment granules exhibit maximal absorption at specific wavelength ranges, thereby serving as spectral filters (Stavenga 1995). Among these, red filtering pigments, characterized by strong absorption of wavelengths shorter than 600 nm, were first identified in the butterfly species *Pieris rapae* (Ribi 1978). Unlike other pupillary pigments in photoreceptors or pigment cells, these red pigment granules do not move substantially in response to light, and are concentrated in clusters in the photoreceptor soma, near the rhabdom. They absorb short-wavelength light as light pass through the rhabdom, a process known as lateral filtering (Ribi 1978). As a result, the presence of red filtering pigments shifts the peak sensitivity of photoreceptors toward longer wavelengths and narrows the sensitivity spectrum, effectively creating distinct long-wavelength photoreceptors. This enables color opponency and finer discrimination across the green-to-red spectrum (Fig. 5).

### The evolution of red photoreceptors in Papilionidae

Papilionidae represents a special case in the evolution of red photoreceptors, characterized by both LW opsin duplications and the presence of red filtering pigments. The duplication of LW opsins enables a broader range of peak spectral sensitivities (Frentiu et al. 2007). Behavioral experiments show that *Papilio xuthus* can discriminate wavelength differences as small as 1 nm at approximately 560 nm (Koshitaka et al. 2008). Even in the red wavelength range around 620 nm, *P. xuthus* can distinguish between different shades of red, although the minimum discriminable wavelength difference increases to 10 nm (Koshitaka et al. 2008).

Four types of filtering pigments have been identified in *P. xuthus* (tribe Papilionini). Each ommatidial type shows a coordinated combination of R1/2 opsin expression and filtering pigments in R1–8. Based on R1/2 opsin expression, the three types are: type I (UV-B), type II (UV-UV), and type III (B-B) (Kitamoto et al. 1998). In the distal region, purple pupillary pigment granules are found in R1/2 cells across all ommatidia. The R3-8 cells of each ommatidium contain clusters of pigment granules, either red (type I and type II) or yellow (type III), located within 1 μm of the rhabdomere boundary. Additionally, type II ommatidia possess UV-absorbing fluorescent pigments, specifically 3-hydroxyretinols. These UV-absorbing pigments modify the spectral sensitivities of UV receptors (R1/2) and double-peak green receptors (R3/4) in type II ommatidia, converting them into narrow-band violet receptors and single-peak green receptors, respectively (Arikawa and Stavenga 1997; Arikawa 2003).

In *P. xuthus*, red-sensitive proximal photoreceptors exhibit a narrow peak at 600 nm, resulting from L3 opsin expression (λ_max_ at 575 nm) combined with red perirhabdomal filtering pigments (Arikawa et al. 1999). Histology studies show that L3 is exclusively expressed in the proximal R5-8 cells of red ommatidia. (Arikawa 2003). This tight association between red filtering pigments and the red-sensitive L3 is also suggested in the distantly-related *Parnassius glacialis* (tribe Parnassiini), where a subset of the ventral ommatidia contain red pigments and express L3 in R3-8 (Awata et al. 2010). In another species, *Troides aeacus formosanus* of the tribe Troidini, a sister tribe to Papilionini, two red receptors (λ_max_ at 610 nm and 630 nm) are found in ommatidia with pale-red and deep-red pigments, respectively (Chen et al. 2013; Condamine et al. 2018). The most striking example of the red receptor diversity is found in *Graphium sarpedon*, a species of the tribe Leptocircini. Electrophysiological recordings reveal five distinct subclasses of red receptors, including a deep-red receptor peaking at 640 nm, which has been histologically identified as the L3a-expressing proximal photoreceptor (Chen et al. 2016).

### The evolution of red photoreceptors in Pieridae

Despite having a single copy of the LW opsin gene, Pieridae butterflies possess some of the most diverse red photoreceptors among Lepidoptera. Similar to *Papilio* butterflies, the rhabdom of Pieridae is fully tiered. In *Colias* butterflies, the rhabdom in ventral ommatidia is divided into proximal and distal tiers by a strong constriction, enhancing the filtering effect of the red perirhabdomal pigments in R5-8 (Arikawa et al. 2009). The most red-shifted green photoreceptor ever recorded in insects is found in *Colias erate*, with a peak sensitivity at 660 nm (Pirih et al. 2010). By varying the spatial distribution of red perirhabdomal pigments and introducing a female-specific orange perirhabdomal pigment, female *C. erate* possess three red photoreceptor types with peak sensitivity at 610 nm, 650 nm, and 660 nm (Ogawa et al. 2013). This expansion pushes their color discrimination range close to the far-red limit of approximately 700 nm. In contrast, male *C. erate* butterflies have only one type of red receptor with peak sensitivity at 660 nm. In the dorsal eye region, which is not sexually dimorphic, R5-8 in both sexes are maximally sensitive at 600–620 nm, due to a moderate filtering effect from lower filtering pigment density and weak constriction (Ogawa et al. 2013). Unlike *C. erate*, both male and female *Pieris rapae* butterflies have three red photoreceptor types in their ventral ommatidia, with peak sensitivities at 610 nm, 630 nm, and 640 nm. These spectral differences arise from the distinct red pigment granules present in each of the three ommatidial types, likely due to varying pigment densities within the granules (Blake et al. 2019).

If all photoreceptors contributed equally to color vision, Pieridae butterflies would be expected to have strong color discrimination in the red range. However, field observations show that neither *Colias* nor *Pieris* butterflies exhibit a preference for red flowers. In a feeding-based behavioral experiment, *P. rapae* butterflies trained on red paper disks preferentially visited orange and purple disks over red, suggesting either poor discrimination within the orange-red spectrum or that red color vision is primarily utilized in non-feeding contexts, such as oviposition (Arikawa et al. 2021).

In addition to red perirhabdomal pigments, *P. rapae* males have a fluorescent pigment in type II ommatidia that emits fluorescence under 420 nm excitation. This pigment turns the violet-sensitive R1/2 photoreceptor into double-peak blue receptors (Qiu et al. 2002; Arikawa et al. 2005). A similar filtering effect occurs in *C. erate* male type I ommatidia and female type II ommatidia (Ogawa et al. 2012).

*Anthocharis* butterflies (subfamily Pierinae) represent a secondary loss of the ommatidial heterogeneity in Pieridae. Only two ommatidial types are distinguishable, based on the arrangement of red perirhabdomal pigments in R5-8. In round-type ommatidia, red pigments are located in the distal half of the ommatidium, whereas in trapezoidal-type ommatidia, they are confined to the proximal third (Takemura et al. 2007).

### The evolution of red photoreceptors in Lycaenidae

Lycaenidae butterflies achieve long-wavelength color vision through a combination of spectral tuning of their B and LW opsins and lateral filtering. The rhabdom structure of Lycaenidae is not fully tiered, based on the electron microscopy study in *Eumaeus atala* (Liénard et al. 2021). R1 and R2 only contribute their microvilli to the distal portion of the rhabdom, while R3-8 contribute the majority of microvilli throughout the rhabdom (Liénard et al. 2021). In *Lycaena rubidus*, a pink filtering pigment is found exclusively in the R5-8 of the ventral eye ommatidia that express B2, a green-shifted B opsin (Sison-Mangus et al. 2006). Across Lycaenidae, many species have also evolved red-shifted LW opsins with peak sensitivities between 564 nm and 571 nm, compared to the ancestral peak near 540 nm (Frentiu et al. 2007; Liénard et al. 2021). In *Polyommatus icarus*, this coordinated shift in B and LW opsins, likely enables them to discriminate color in the green wavelength range, up to 560 nm. However, behavioral experiments show that *P. icarus* cannot differentiate colors in the red range (570–640 nm), indicating that their long-wavelength color vision does not extend into the true red spectrum (Sison-Mangus et al. 2008). One possible explanation is the absence of pink filtering pigments in the distal ommatidia, which reduces spectral filtering for LW photoreceptors and consequently limits their sensitivity in the red spectrum (Sison-Mangus et al. 2006).

### The evolution of red photoreceptors in Nymphalidae

Although most Nymphalidae species possess only one LW opsin and one B opsin, true red color vision has been verified through behavioral experiments in nymphalid species *Heliconius erato* (Zaccardi et al. 2006) and *Danaus plexippus* (Blackiston et al. 2011). In *Heliconius*, two types of filtering pigments have been identified, with peak absorbance at approximately 450 nm and 560 nm. The red pigment (λ_max_ at 560 nm) is likely ommin, a type of sulfur-containing ommochrome commonly found in insect eyes (Langer and Struwe 1972). The presence of these red filtering pigments is closely associated with the presence of red-sensitive photoreceptors in Nymphalidae.

**Fig. 6:**
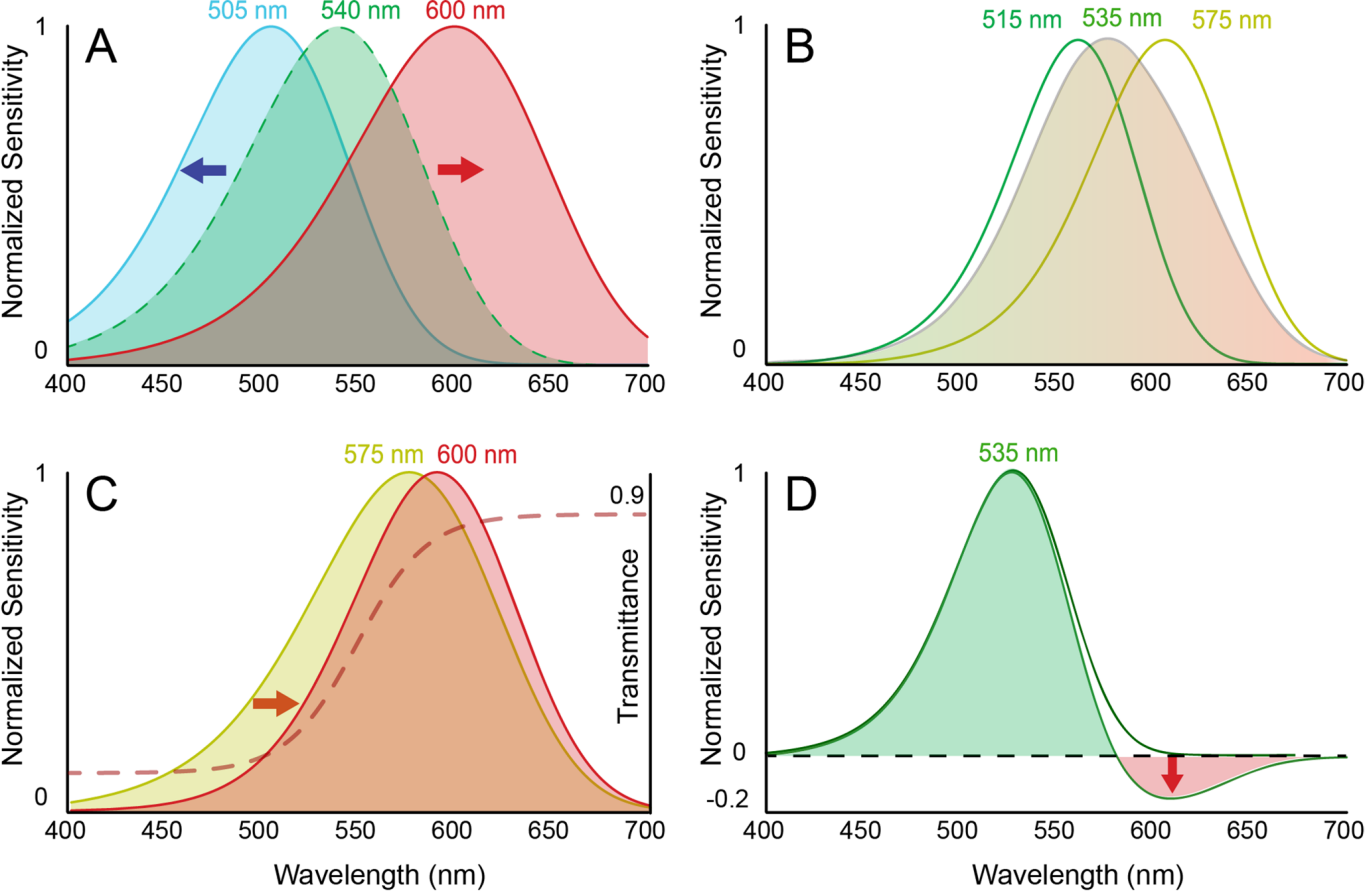
Mechanisms that modify photoreceptor spectral sensitivity. **A** *Gene duplication and divergence.* In *Apodemia mormo*, an ancestral LW opsin underwent duplication. The two resulting copies have since accumulated amino acid substitutions, producing a red-shifted opsin and a blue-shifted opsin (Frentiu et al. 2007). **B** *Opsin co-expression.* In *Papilio xuthus*, co-expression of opsins L2 and L3 generates a broadband photoreceptor with peak sensitivity around 535 nm (Arikawa et al. 2003). **C** *Lateral filtering.* In *Papilio xuthus*, the proximal R5–8 photoreceptors in type I ommatidia express L3 (λ_max_ 575 nm). The red filtering pigment acts as a short-wavelength absorbing filter that reduces sensitivity in the short wavelength range, thereby narrowing the bandwidth and shifting the peak sensitivity to 600 nm. The red dashed line represents the transmittance curve of the red filtering pigment, which is nearly transparent above 580 nm (Arikawa et al. 1999). **D** *Direct inhibition.* In *Charaxes jasius*, green photoreceptors that receive direct inhibitory input from red photoreceptors (λmax 620 nm) retain their peak sensitivity at 535 nm, but display a narrower spectral bandwidth and a hyperpolarizing response in the red wavelength region (Belušič et al. 2021). The figure displays only the α-bands. The β-bands, which are typically present, have been omitted for clarity.

A novel class of green-sensitive photoreceptors that hyperpolarize in response to red light (Fig. 6) has been identified across multiple Nymphalidae subfamilies (Belušič et al. 2021). These green-positive, red-negative (G + R-) cells have been allocated to the R1/2 positions and are observed exclusively in species with red eyeshine, which indicates the presence of red filtering pigments (Belušič et al. 2021). The presence of G + R- R1/2 photoreceptors expands the retinal mosaic from a simple arrangement of three ommatidial types (based on B and UV R1/2) to a complex pattern comprising six distinct ommatidial types (Pirih et al. 2022). Co-expression of LW and B opsins in R1/2 photoreceptors has been detected throughout the Heliconiini clade using antibody staining (McCulloch et al. 2022b; Chakraborty et al. 2023). These cells likely correspond to the G + R- photoreceptors involved in red-green color opponency. Within this circuit, the red opponent units (R–) are thought to be the basal photoreceptors R9 (Belušič et al. 2021; Ilić et al. 2022; Pirih et al. 2022). While red-sensitive photoreceptors have been directly recorded in multiple *Heliconius* species (McCulloch et al. 2017, 2022b; VanKuren et al. 2025), the precise identity of these recorded red receptors (whether they correspond to the R9 cell or R3–8 cells) remains unconfirmed.

This R9 localization of red receptors represents a striking contrast to the R3-8 red receptors found in Papilionidae and Pieridae (Fig. 5). The rhabdom in nymphalids is not fully tiered (Kolb 1985), with R3-8 contributing microvilli throughout much of the rhabdom, potentially making R9 better suited to receive light filtered by red pigments. Despite extensive characterization of R1/2-based ommatidial types in *Heliconius*, the relationship between R1/2 opsin expression and the presence of red filtering pigments remains unresolved (Buerkle et al. 2022). One hypothesis based on electrophysiological data is that broadband green R1/2 photoreceptors are restricted to red-reflecting ommatidia, but histology studies are needed to confirm this association.

Complex retinal mosaics with red-reflecting ommatidia are found in both sexes of many nymphalids, including early-diverging Danaini (Blackiston et al. 2011). However, the red perirhabdomal pigments have been lost multiple times in Nymphalini (Briscoe and Bernard 2005) and Apaturini (Pirih et al. 2022), which retain ancestral trichromatic color vision, consisting of UV-, blue- and green-sensitive photoreceptors. In Argynnini butterflies, females have secondarily lost the red-reflecting ommatidia, while males retain an expanded retinal mosaic with red-sensitive photoreceptors (Ilić et al. 2022). Overall, the gain and loss of red lateral filtering pigments appear to be highly evolutionarily labile.

## Regional differences and visual ecology

Dorsal-ventral variation in the compound eye is common across Lepidoptera. In many species, the dorsal region of the eye retains a more conserved and likely ancestral arrangement of ommatidia, characterized by fewer ommatidial types and the absence of fluorescent or perirhabdomal filtering pigments (Qiu and Arikawa 2003; Awata et al. 2010; Ogawa et al. 2013; Chen et al. 2016). The dorsal and ventral regions of the eye can also differ structurally. In *Leptidea amurensis*, the ventral eye exhibits a distinctive rough appearance caused by an irregular distribution of facets in two distinct sizes (Uchiyama et al. 2013). The most extreme example of this is found in the hawkmoth *Manduca sexta*, where the dorsal ommatidia structurally resemble those of ancestral winged insects with only a single dR7 cell (White et al. 2003; Gao et al. 2025). These differences between ventral and dorsal eye regions likely reflect their distinct roles in visual ecology. The ventral eye region is thought to be important for behaviors such as host plant recognition and mate detection, while the dorsal eye may be more important for predator detection. However, exceptions exist. In highly territorial *Lycaenae* butterflies, the dorsal region is sexually dimorphic. Males express B1 opsins in R3-8 photoreceptors, which may enhance their ability to detect rival, conspecific males (Sison-Mangus et al. 2006).

In many insects, including Lepidoptera, ommatidia in a small region of the compound eye, known as the dorsal rim area (DRA), are anatomically specialized for detecting polarized skylight (Labhart and Meyer 1999, 2002). Although debated, detection of polarized UV light may play an important role in flight orientation in monarch butterflies (*Danaus plexippus*), which are renowned for their long-distance migration (Sauman et al. 2005; Stalleicken et al. 2005). In the monarch butterfly, each DRA ommatidium contains two anatomical types of photoreceptors with mutually orthogonal microvilli, providing the basis for polarization antagonism (Reppert et al. 2004). To avoid interference with color information, R1-8 in monarch DRA ommatidia express UV opsins exclusively (Sauman et al. 2005). Additionally, the monarch DRA lacks functional tapeta found in other parts of the eye (Labhart et al. 2009).

Compared to the monarch, which has approximately 100 ommatidia in the DRA, the nocturnal hawkmoth *Manduca sexta* has a much larger DRA containing around 1,000 ommatidia (White et al. 2003). Such an extensive DRA is also observed in other nocturnal moth species and may play an important role in navigation under dim light (Meinecke 1981; Anton-Erxleben and Langer 1988; Belušič et al. 2017). In *M. sexta*, the tapetum in the DRA is also greatly reduced, enveloping only the proximal ends of the photoreceptors, likely to enable a larger visual field. Only a subset of R1/2 in DRA ommatidia express UV opsin, while the remaining R1/2 and all R3–8 lack expression of UV, B, or LW opsins (White et al. 2003). In the European corn borer moth, *Ostrinia nubilalis*, photoreceptors in the DRA express B or LW opsins (Belušič et al. 2017).

Interestingly, highly polarization-sensitive photoreceptors have also been found outside the DRA. In *O. nubilalis*, distal blue-sensitive R1/2 photoreceptors in the main retina exhibit stronger polarization sensitivity than photoreceptors in the DRA (Belušič et al. 2017). Similar polarization-sensitive ommatidia outside the DRA have also been observed in *Drosophila* where they may play a role in sensing the reflection from water (Wernet et al. 2012).

## Molecular logic underlying diverse retinal mosaics

Comprehensive reviews on retinal mosaics across insects are available in (Wernet et al. 2015; McCulloch et al. 2022a). Here, we highlight the unique challenges and opportunities in uncovering the molecular logic that shapes the retinal mosaic in butterflies and moths. In *Papilio xuthus*, previous studies have shown that two independent stochastic decisions regarding expression of the transcription factor *spineless* in R1/2 photoreceptors give rise to three ommatidial types (Perry et al. 2016). This mechanism is similar to the pale vs. yellow ommatidial fate decision in *Drosophila* (Wernet et al. 2006). Stochastic *spineless* expression not only determines the opsin identity in R1/2 (B or UV) but also coordinates other features of the whole ommatidium, including LW opsin expression in R3-8 and the presence of red perirhabdomal or fluorescent filtering pigments (Perry et al. 2016). This tight coordination of filtering pigments and opsins across all photoreceptors within an ommatidium is likely crucial for efficient downstream visual processing, as axons of all nine photoreceptors from the same ommatidium project through the same cartridge in the lamina (Matsushita et al. 2022).

In *Heliconius* and other Nymphalidae butterflies, the presence of red filtering pigments and broadband green-sensitive R1/2 cells results in at least six types of ommatidia. However, the underlying logic generating this expanded retinal mosaic remains unclear. A simple three-way stochastic choice of broadband/UV/B photoreceptors cannot explain the relative proportion of UV-B, B-B, and UV-UV observed. Furthermore, in females of the *Heliconius erato/sara*/*sapho* clade, this complexity is increased by an additional stochastic choice between UV1 or UV2 in R1/2.

The stochastic expression of *spineless* can be modified regionally to generate dorsal-ventral specialization. In *Drosophila*, for example, the dorsal third of the retina contains yellow dR7 cells co-expressing Rh3 and Rh4, which are typically restricted to expressing only Rh4 (Mazzoni et al. 2008). This co-expression is driven by reduced inhibition from lower *spineless* expression and activation from the Iroquois complex transcription factors (Mazzoni et al. 2008; Thanawala et al. 2013). The *Lycaena* butterflies, with both dorsal-ventral retinal specialization and sexually dimorphic dorsal eyes, represent promising candidates to test the role of *spineless* and the Iroquois complex in regional specialization in the context of sexual dimorphism (Sison-Mangus et al. 2006). Dorsal–ventral differences in the distribution of filtering pigments are widespread in butterflies. Investigating how filtering pigments are regulated during dorsal–ventral patterning, and comparing these processes to pigment regulation in stochastic ommatidial differentiation, may provide insights into broader mechanisms of tissue patterning.

## Spectral sensitivity and color vision

Photoreceptor spectral sensitivity is shaped by a combination of molecular and optical features (Fig. 6), including opsin gene duplication and divergence, spectral tuning of opsin protein sequences, co-expression of multiple opsins, and lateral filtering by perirhabdomal pigments (van der Kooi et al. 2021; Mulhair et al. 2023). In many butterfly lineages, these mechanisms combine to produce extreme photoreceptor diversity (Arikawa et al. 1987; Ogawa et al. 2013; Chen et al. 2013, 2016; McCulloch et al. 2017; Blake et al. 2019). For example, in *Graphium sarpedon*, as many as 15 distinct spectral sensitivities have been identified due to a combination of multiple opsin duplications and distinct lateral filtering pigments (Pirih et al. 2022).

Color vision depends on both photoreceptor diversity and the neural circuits that compare signals from these diverse photoreceptors (Schnaitmann et al. 2020). Such comparisons are encoded by color-opponent neurons that exhibit excitation at certain wavelengths and inhibition at others. In *Drosophila*, color-opponent processing occurs as early as the photoreceptor stage, where direct inhibitory synapses only form between the long visual fibers of dR7 and dR8 photoreceptors in optic chiasm or medulla (Schnaitmann et al. 2018; Kind et al. 2021). In *Papilio* butterflies, however, extensive inter-photoreceptor inhibitions exist among long visual fibers (R1/2) and short visual fibers (R3-8 and R9) within the lamina, contributing to the spectrally complex visual system (Matsushita et al. 2022). These photoreceptors with spectral opponency have also been recorded in other Papilionidae and Nymphalidae species (Chen et al. 2013, 2020a; Belušič et al. 2021; Ilić et al. 2022; Pirih et al. 2022; VanKuren et al. 2025).

Despite the high diversity of photoreceptor types in Lepidoptera, not all contribute to color opponency or color vision at the same time. The minimum discriminable wavelength difference function of foraging *Papilio xuthus* exhibits three minima, indicating that only four classes of receptors contribute to color vision (tetrachromatic), despite the presence of at least eight distinct spectral sensitivity types (Koshitaka et al. 2008). The photoreceptors not contributing to tetrachromacy during foraging are all confined to type II ommatidia (Koshitaka et al. 2008). These excluded photoreceptors are likely specialized for non-chromatic functions such as motion detection or polarization vision, or they may be involved in color vision for mating or oviposition. For instance, R3/4 in *P. xuthus* exhibit the fastest response latencies among photoreceptors (Kawasaki et al. 2015), a characteristic that may facilitate motion detection using chromatic contrast (Stewart et al. 2015).

## Non-photoreceptor cells in compound eye

Retinal development has been well characterized in *Drosophila melanogaster*, where the adult compound eye arises from a monolayer of undifferentiated epithelium known as the eye-antennal disc (Kumar 2012). During larval and pupal stages, photoreceptor neurons are specified first, followed by the recruitment of cone cells and primary pigment cells. Cells that do not adopt one of these fates subsequently differentiate into secondary or tertiary pigment cells (Kumar 2012). In Lepidoptera, retinal development follows a similar sequence, at least for photoreceptor recruitment (Monsma and Booker 1996; Gao et al. 2025). Our current understanding of non-photoreceptor cells in Lepidoptera is primarily based on ultrastructure studies using electron microscopy.

Each ommatidium typically has four cone cells, two primary pigment cells (PPCs), and six secondary pigment cells (SPCs), which are shared between adjacent ommatidia (Ribi 1978; Kolb 1985). Cone cells secrete the dioptric apparatus, including cornea and crystalline cone. In *Drosophila*, they can also direct cell type differentiation during ommatidia development and support homeostasis in adult photoreceptors (Charlton-Perkins et al. 2017, 2021). In *Pieris*, the PPCs envelop the cone cells and the distal half of the crystalline cone. These pigment cells help regulate light influx by contracting distally during light adaptation. SPCs cover the proximal half of the crystalline cone and the photoreceptors down to the basement membrane, shielding stray light from nearby ommatidia. During light adaptation, pigment granules in SPCs accumulate in the distal region. Another set of pigment cells, basal pigment cells (BPCs), are located below the basement membrane. These cells insulate photoreceptor axons and, together with SPCs, form the dense pigmentation layer at the base of the ommatidium (Ribi 1978). BPC pigment granules differ significantly in size compared to those of PPCs, SPCs, and photoreceptors (Fischer et al. 2012). This suggests that the subretinal pigment layer consists of a novel pigment cell type. In other insects, the subretinal pigment layer is derived from secondary/tertiary pigment cells (Tomlinson 2012) or lateral rim pigment cells (Mohr et al. 2020). Whether BPCs originate from subretinal or retinal tissue remains unclear. Comparative transcriptomic analysis with PPCs, SPCs, and other subretinal glial cells may help resolve their developmental origin.

In Lepidoptera, tracheal cells form the tapetum at the base of the rhabdom. In nocturnal moths, the tapetum consists of numerous tracheoles with alternating air and cytoplasm, forming an interference reflector that mirrors unabsorbed light back through the rhabdom. The tapetum is located just above the basement membrane. In many diurnal butterflies, this ancestral tracheal tapetum has been modified into a few branches at the proximal end of the rhabdom (Ribi 1979). The tapetum has been independently lost at least three times in butterflies: once at the base of the Papilionidae family, and twice within Pieridae, specifically in *Leptidea amurensis* and *Anthocharis scolymus* (Takemura et al. 2007; Uchiyama et al. 2013).

## Conclusions and future directions

The compound eyes of butterflies and moths exhibit remarkable diversity in cellular composition, spectral tuning, and spatial organization. The spectral diversity arises from a combination of opsin gene duplication and divergence, opsin co-expression, lateral filtering, and direct inhibition among photoreceptors. Different lineages, such as Papilionidae, Lycaenidae, and Nymphalidae, have evolved distinct mechanisms to expand sensitivity into the long-wavelength range, through a combination of red filtering pigments, LW opsin duplication and divergence, and green-shifted B opsin. These innovations, along with regional specializations like dorsal–ventral patterning and polarization-sensitive DRA ommatidia, reflect tight coordination among anatomical, molecular, and functional components of the eye. For example, in *Papilio xuthus*, five opsins and three types of filtering pigments are integrated into just three ommatidial types. We have only begun to understand the developmental mechanisms that underlie this precisely regulated coordination of the various features of ommatidia (Perry et al. 2016).

Despite progress in characterizing eye structure and photoreceptor diversity, many fundamental questions remain. The molecular logic underlying complex retinal mosaics, particularly in species with more than three ommatidial types like *Heliconius*, is still unknown. Similarly, the developmental origin and function of lesser-known cell types, such as basal pigment cells, and the role of photoreceptors not involved in color vision remain poorly understood. These photoreceptors may contribute to spatial and motion vision, or even wavelength-specific behaviors that are ecologically important but largely unexplored. Additionally, the rapid turnover in eye designs across Lepidoptera, including repeated shifts between apposition and superposition eyes, raises questions about how intermediate forms remain functional. Understanding the impact of opsin or filtering pigment changes on downstream visual circuits will be key to understanding how color processing is preserved or reshaped during evolution.

To address these gaps, future research should focus on three main areas. First, identifying the ecological pressures that drive visual system diversification will clarify the adaptive value of specific photoreceptor types and spectral sensitivities. Second, dissecting the genetic and regulatory basis of compound eye variation, especially with the help of comparative genomics and single-cell multi-omics, will illuminate how new eye designs evolve and what constraints shape them. Finally, much remains to be learned about visual processing circuits in butterflies and moths, particularly given their unique features like the multi-tiered rhabdom structure, diverse opsin and pigment variation in R1-8, and the R9 photoreceptor projecting to the lamina (Matsushita et al. 2022). Understanding how these differences influence color perception and behavior will provide deeper insight into the evolution of sensory systems more broadly.

## Supplementary Information

Below is the link to the electronic supplementary material.

Supplementary material 1 (XLSX 43.5 kb)

## Data Availability

No datasets were generated or analysed during the current study.

## References

[CR1] Anton-Erxleben F, Langer H (1988) Functional morphology of the ommatidia in the compound eye of the moth, Antheraea polyphemus (Insecta, Saturniidae). Cell Tissue Res 252:385–396. 10.1007/BF00214381 3383218 10.1007/BF00214381

[CR2] Arikawa K (2003) Spectral organization of the eye of a butterfly, Papilio. J Comp Physiol Sens Neural Behav Physiol 189:791–800. 10.1007/s00359-003-0454-7 10.1007/s00359-003-0454-7 14520495

[CR3] Arikawa K (2017) The eyes and vision of butterflies. J Physiol 595:5457–5464. 10.1113/JP273917 28332207 10.1113/JP273917 PMC5556174

[CR4] Arikawa K, Stavenga DG (1997) Random array of colour filters in the eyes of butterflies. J Exp Biol 200:2501–2506. 10.1242/jeb.200.19.2501 9320430 10.1242/jeb.200.19.2501

[CR5] Arikawa K, Uchiyama H (1996) Red receptors dominate the proximal tier of the retina in the butterfly Papilio xuthus. J Comp Physiol A 178:55–61. 10.1007/BF00189590

[CR6] Arikawa K, Inokuma K, Eguchi E (1987) Pentachromatic visual system in a butterfly. Naturwissenschaften 74:297–298. 10.1007/BF00366422

[CR7] Arikawa K, Scholten DGW, Kinoshita M, Stavenga DG (1999) Tuning of photoreceptor spectral sensitivities by red and yellow pigments in the butterfly Papilio xuthus. Zoolog Sci 16:17–24. 10.2108/zsj.16.17

[CR8] Arikawa K, Mizuno S, Kinoshita M, Stavenga DG (2003) Coexpression of two visual pigments in a photoreceptor causes an abnormally broad spectral sensitivity in the eye of the butterfly Papilio xuthus. J Neurosci 23:4527–4532. 10.1523/JNEUROSCI.23-11-04527.2003 12805293 10.1523/JNEUROSCI.23-11-04527.2003 PMC6740815

[CR9] Arikawa K, Wakakuwa M, Qiu X et al (2005) Sexual dimorphism of Short-Wavelength photoreceptors in the small white butterfly, Pieris rapae crucivora. J Neurosci 25:5935–5942. 10.1523/JNEUROSCI.1364-05.2005 15976082 10.1523/JNEUROSCI.1364-05.2005 PMC6724796

[CR10] Arikawa K, Pirih P, Stavenga DG (2009) Rhabdom constriction enhances filtering by the red screening pigment in the eye of the Eastern pale clouded yellow butterfly, Colias erate (Pieridae). J Exp Biol 212:2057–2064. 10.1242/jeb.030692 19525432 10.1242/jeb.030692

[CR11] Arikawa K, Iwanaga T, Wakakuwa M, Kinoshita M (2017) Unique Temporal expression of triplicated Long-Wavelength Opsins in developing butterfly eyes. Front Neural Circuits 11:96. 10.3389/fncir.2017.00096 29238294 10.3389/fncir.2017.00096 PMC5712540

[CR12] Arikawa K, Nakatani Y, Koshitaka H, Kinoshita M (2021) Foraging small white butterflies, Pieris rapae, search flowers using color vision. Front Ecol Evol 9:650069. 10.3389/fevo.2021.650069

[CR13] Awata H, Wakakuwa M, Arikawa K (2009) Evolution of color vision in Pierid butterflies: blue Opsin duplication, ommatidial heterogeneity and eye regionalization in Colias erate. J Comp Physiol A 195:401–408. 10.1007/s00359-009-0418-7 10.1007/s00359-009-0418-7 19224222

[CR14] Awata H, Matsushita A, Wakakuwa M, Arikawa K (2010) Eyes with basic dorsal and specific ventral regions in the glacial apollo, Parnassius glacialis (Papilionidae). J Exp Biol 213:4023–4029. 10.1242/jeb.048678 21075944 10.1242/jeb.048678

[CR15] Bandai K, Arikawa K, Eguchi E (1992) Localization of spectral receptors in the Ommatidium of butterfly compound eye determined by polarization sensitivity. J Comp Physiol A 171:289–297. 10.1007/BF00223959

[CR16] Belušič G, Šporar K, Meglič A (2017) Extreme polarization sensitivity in the retina of the corn borer moth Ostrinia. J Exp Biol 220:2047–2056. 10.1242/jeb.153718 28341662 10.1242/jeb.153718

[CR17] Belušič G, Ilić M, Meglič A, Pirih P (2021) Red-green opponency in the long visual fibre photoreceptors of brushfoot butterflies (Nymphalidae). Proc R Soc B. 10.1098/RSPB.2021.1560 34702070 10.1098/rspb.2021.1560 PMC8548807

[CR18] Bernard GD, Remington CL (1991) Color vision in Lycaena butterflies: spectral tuning of receptor arrays in relation to behavioral ecology. Proc Natl Acad Sci 88:2783–2787. 10.1073/pnas.88.7.2783 2011588 10.1073/pnas.88.7.2783 PMC51323

[CR19] Blackiston D, Briscoe AD, Weiss MR (2011) Color vision and learning in the monarch butterfly, Danaus plexippus (Nymphalidae). J Exp Biol 214:509–520. 10.1242/jeb.048728 21228210 10.1242/jeb.048728

[CR20] Blake AJ, Pirih P, Qiu X et al (2019) Compound eyes of the small white butterfly Pieris rapae have three distinct classes of red photoreceptors. J Comp Physiol A 205:553–565. 10.1007/s00359-019-01330-8 10.1007/s00359-019-01330-8 31123814

[CR21] Briscoe AD (2000) Six Opsins from the butterfly Papilio glaucus: molecular phylogenetic evidence for paralogous origins of Red-Sensitive visual pigments in insects. J Mol Evol 51:110–121. 10.1007/s002390010071 10948267 10.1007/s002390010071

[CR22] Briscoe AD (2008) Reconstructing the ancestral butterfly eye: focus on the Opsins. J Exp Biol 211:1805–1813. 10.1242/jeb.013045 18490396 10.1242/jeb.013045

[CR23] Briscoe AD, Bernard GD (2005) Eyeshine and spectral tuning of long wavelength-sensitive rhodopsins: no evidence for red-sensitive photoreceptors among five Nymphalini butterfly species. J Exp Biol 208:687–696. 10.1242/jeb.01453 15695761 10.1242/jeb.01453

[CR24] Briscoe AD, Chittka L (2001) The evolution of color vision in insects. Annu Rev Entomol 46:471–510. 10.1146/annurev.ento.46.1.471 11112177 10.1146/annurev.ento.46.1.471

[CR25] Briscoe AD, Bernard GD, Szeto AS et al (2003) Not all butterfly eyes are created equal: rhodopsin absorption spectra, molecular identification, and localization of ultraviolet-, blue‐, and green‐sensitive rhodopsin‐encoding mRNAs in the retina of Vanessa cardui. J Comp Neurol 458:334–349. 10.1002/cne.10582 12619069 10.1002/cne.10582

[CR26] Briscoe AD, Bybee SM, Bernard GD et al (2010) Positive selection of a duplicated UV-sensitive visual pigment coincides with wing pigment evolution in Heliconius butterflies. Proc Natl Acad Sci U S A 107:3628–3633 20133601 10.1073/pnas.0910085107 PMC2840532

[CR27] Chakraborty M, Lara AG, Dang A et al (2023) Sex-linked gene traffic underlies the acquisition of sexually dimorphic UV color vision in Heliconius butterflies. Proc Natl Acad Sci 120:e2301411120. 10.1073/pnas.2301411120 37552755 10.1073/pnas.2301411120 PMC10438391

[CR28] Champlin DT, Truman JW (1998) Ecdysteroids govern two phases of eye development during metamorphosis of the moth, Manduca sexta. Development 125:2009–2018. 10.1242/dev.125.11.2009 9570766 10.1242/dev.125.11.2009

[CR29] Charlton-Perkins MA, Sendler ED, Buschbeck EK, Cook TA (2017) Multifunctional glial support by semper cells in the Drosophila retina. PLOS Genet 13:e1006782. 10.1371/journal.pgen.1006782 28562601 10.1371/journal.pgen.1006782 PMC5470715

[CR30] Charlton-Perkins MA, Friedrich M, Cook TA (2021) Semper’s cells in the insect compound eye: insights into ocular form and function. Dev Biol 479:126–138. 10.1016/J.YDBIO.2021.07.015 34343526 10.1016/j.ydbio.2021.07.015 PMC8410683

[CR31] Chazot N, Wahlberg N, Freitas AVL et al (2019) Priors and posteriors in bayesian timing of divergence analyses: the age of butterflies revisited. Syst Biol 68:797–813. 10.1093/sysbio/syz002 30690622 10.1093/sysbio/syz002 PMC6893297

[CR32] Chen P-J, Arikawa K, Yang E-C (2013) Diversity of the photoreceptors and spectral opponency in the compound eye of the golden birdwing, troides Aeacus formosanus. PLoS ONE 8:e62240. 10.1371/journal.pone.0062240 23614043 10.1371/journal.pone.0062240 PMC3627921

[CR33] Chen P-J, Awata H, Matsushita A et al (2016) Extreme spectral richness in the eye of the common Bluebottle butterfly, graphium sarpedon. Front Ecol Evol 4:177822. 10.3389/fevo.2016.00018

[CR34] Chen P-J, Belušič G, Arikawa K (2020a) Chromatic information processing in the first optic ganglion of the butterfly Papilio xuthus. J Comp Physiol A 206:199–216. 10.1007/s00359-019-01390-w 10.1007/s00359-019-01390-w PMC7069911 31838572

[CR35] Chen Z, Niu Y, Liu C-Q, Sun H (2020b) Red flowers differ in shades between pollination systems and across continents. Ann Bot 126:837–848. 10.1093/aob/mcaa103 32478385 10.1093/aob/mcaa103 PMC7539362

[CR36] Condamine FL, Nabholz B, Clamens AL et al (2018) Mitochondrial phylogenomics, the origin of swallowtail butterflies, and the impact of the number of clocks in bayesian molecular dating. Syst Entomol 43:460–480. 10.1111/syen.12284

[CR37] Dang A, Bernard GD, Yuan F et al (2025) Trichromacy is insufficient for mate detection in a mimetic butterfly. Commun Biol 8:189. 10.1038/s42003-025-07472-7 39915690 10.1038/s42003-025-07472-7 PMC11802900

[CR38] Dell’Angelica EC, Mullins C, Caplan S, Bonifacino JS (2000) Lysosome-related organelles. FASEB J 14:1265–1278. 10.1096/fasebj.14.10.1265 10877819 10.1096/fj.14.10.1265

[CR39] Dietrich W (1909) Die Facettenaugen der dipteren. Z Für Wissenschaftliche Zool 92:465–539

[CR40] Domazet-Lošo T, Tautz D (2010) A phylogenetically based transcriptome age index mirrors ontogenetic divergence patterns. Nature 468:815–818. 10.1038/nature09632 21150997 10.1038/nature09632

[CR41] Kuwalekar M, Deshmukh R, Baral S et al (2022) Duplication and sub-functionalisation characterise diversification of Opsin genes in the Lepidoptera. bioRxiv 2022.10.31.514481

[CR42] Eguchi E, Watanabe K, Hariyama T, Yamamoto K (1982) A comparison of electrophysiologically determined spectral responses in 35 species of Lepidoptera. J Insect Physiol 28:675–682. 10.1016/0022-1910(82)90145-7

[CR43] Ehrlich PR, Raven PH (1964) BUTTERFLIES AND PLANTS: A STUDY IN COEVOLUTION. Evol (N Y) 18:586–608. 10.1111/j.1558-5646.1964.tb01674.x

[CR44] Espeland M, Breinholt J, Willmott KR et al (2018) A comprehensive and dated phylogenomic analysis of butterflies. Curr Biol 28:770–778e5. 10.1016/j.cub.2018.01.061 29456146 10.1016/j.cub.2018.01.061

[CR45] Exner S (1891) Die physiologie der facettirten augen von Krebsen und Insecten. Franz Deuticke, Leipzig

[CR46] Finkbeiner SD, Briscoe AD (2021) True UV color vision in a female butterfly with two UV opsins. J Exp Biol. 10.1242/jeb.242802 34587624 10.1242/jeb.242802

[CR47] Fischer S, Müller CHG, Meyer-Rochow VB (2012) Neither apposition nor superposition: the compound eyes of the chestnut leafminer cameraria ohridella. Zoomorphology 131:37–55. 10.1007/s00435-011-0141-0

[CR48] Fischer S, Meyer-Rochow VB, Müller CHG (2014) Compound eye miniaturization in lepidoptera: A comparative morphological analysis. Acta Zool 95:438–464. 10.1111/azo.12041

[CR49] Frentiu FD, Bernard GD, Sison-Mangus MP et al (2007) Gene duplication is an evolutionary mechanism for expanding spectral diversity in the Long-Wavelength photopigments of butterflies. Mol Biol Evol 24:2016–2028. 10.1093/molbev/msm132 17609538 10.1093/molbev/msm132

[CR50] Friedrich M, Wood EJ, Wu M (2011) Developmental evolution of the insect retina: insights from standardized numbering of homologous photoreceptors. J Exp Zool Part B Mol Dev Evol 316B:484–499. 10.1002/jez.b.21424 10.1002/jez.b.21424 21796775

[CR51] Gao K, Donati A, Ainsworth J et al (2025) Deep conservation complemented by novelty and innovation in the insect eye ground plan. Proc Natl Acad Sci 122:e2416562122. 10.1073/pnas.2416562122 39793041 10.1073/pnas.2416562122 PMC11725883

[CR52] Gordon WC (1977) Microvillar orientation in the retina of the nymphalid butterfly. Z Für Naturforsch C 32:662–664. 10.1515/znc-1977-7-833

[CR53] Harzsch S, Hafner G (2006) Evolution of eye development in arthropods: phylogenetic aspects. Arthropod Struct Dev 35:319–340. 10.1016/j.asd.2006.08.009 18089079 10.1016/j.asd.2006.08.009

[CR54] Heikkilä M, Kaila L, Mutanen M et al (2012) Cretaceous origin and repeated tertiary diversification of the redefined butterflies. Proc R Soc B Biol Sci 279:1093–1099. 10.1098/rspb.2011.1430 10.1098/rspb.2011.1430 PMC3267136 21920981

[CR55] Henze MJ, Oakley TH (2015) The dynamic evolutionary history of pancrustacean eyes and Opsins. Integr Comp Biol 55:830–842. 10.1093/icb/icv100 26319405 10.1093/icb/icv100

[CR56] Hirota SK, Nitta K, Suyama Y et al (2013) Pollinator-Mediated selection on flower color, flower scent and flower morphology of hemerocallis: evidence from genotyping individual pollen grains on the stigma. PLoS ONE 8:e85601. 10.1371/journal.pone.0085601 24376890 10.1371/journal.pone.0085601 PMC3871637

[CR57] Honkanen A, Meyer-Rochow VB (2009) The eye of the parthenogenetic and minute moth ectoedemia argyropeza (Lepidoptera: Nepticulidae). Eur J Entomol 106:619–629. 10.14411/eje.2009.078

[CR58] Horridge GA, Giddings C (1971) The retina of ephestia (Lepidoptera). Proc R Soc Lond Ser B Biol Sci 179:87–95. 10.1098/rspb.1971.0083

[CR59] Horridge GA, McLean M, Stange G, Lillywhite PG (1977) A diurnal moth superposition eye with high resolution phalaenoides tristifica (Agaristidae). Proc R Soc Lond Ser B Biol Sci 196:233–250. 10.1098/rspb.1977.0039 16267 10.1098/rspb.1977.0039

[CR60] Hu X, Whaley MA, Stein MM et al (2011) Coexpression of spectrally distinct rhodopsins in Aedes aegypti R7 photoreceptors. PLoS ONE 6:e23121. 10.1371/journal.pone.0023121 21858005 10.1371/journal.pone.0023121 PMC3152566

[CR61] Ilić M, Chen P-J, Pirih P et al (2022) Simple and complex, sexually dimorphic retinal mosaic of fritillary butterflies. Philos Trans R Soc B Biol Sci. 10.1098/rstb.2021.0276 10.1098/rstb.2021.0276 PMC9441240 36058236

[CR62] Johnson SD, Bond WJ (1994) Red flowers and butterfly pollination in the fynbos of South Africa. Springer, Dordrecht, pp 137–148

[CR63] Kalinka AT, Varga KM, Gerrard DT et al (2010) Gene expression divergence recapitulates the developmental hourglass model. Nature 468:811–814. 10.1038/nature09634 21150996 10.1038/nature09634

[CR64] Kawahara AY, Plotkin D, Hamilton CA et al (2018) Diel behavior in moths and butterflies: a synthesis of data illuminates the evolution of Temporal activity. Org Divers Evol 18:13–27. 10.1007/s13127-017-0350-6

[CR65] Kawahara AY, Plotkin D, Espeland M et al (2019) Phylogenomics reveals the evolutionary timing and pattern of butterflies and moths. Proc Natl Acad Sci 116:22657–22663. 10.1073/pnas.1907847116 31636187 10.1073/pnas.1907847116 PMC6842621

[CR66] Kawahara AY, Storer C, Carvalho APS et al (2023) A global phylogeny of butterflies reveals their evolutionary history, ancestral hosts and biogeographic origins. Nat Ecol Evol 7:903–913. 10.1038/s41559-023-02041-9 37188966 10.1038/s41559-023-02041-9 PMC10250192

[CR67] Kawasaki M, Kinoshita M, Weckström M, Arikawa K (2015) Difference in dynamic properties of photoreceptors in a butterfly, Papilio xuthus: possible segregation of motion and color processing. J Comp Physiol A 201:1115–1123. 10.1007/s00359-015-1039-y 10.1007/s00359-015-1039-y 26329322

[CR68] Kelber A (1999) Ovipositing butterflies use a red receptor to see green. J Exp Biol 202:2619–2630. 10.1242/jeb.202.19.2619 10482721 10.1242/jeb.202.19.2619

[CR69] Kelber A, Balkenius A, Warrant EJ (2002) Scotopic colour vision in nocturnal hawkmoths. Nature 419:922–925. 10.1038/nature01065 12410310 10.1038/nature01065

[CR70] Kelber A, Balkenius A, Warrant EJ (2003) Colour vision in diurnal and nocturnal hawkmoths. Integr Comp Biol 43:571–579. 10.1093/icb/43.4.571 21680465 10.1093/icb/43.4.571

[CR71] Kiepiel I, Johnson SD (2014) Shift from bird to butterfly pollination in Clivia (Amaryllidaceae). Am J Bot 101:190–200. 10.3732/ajb.1300363 24414430 10.3732/ajb.1300363

[CR72] Kind E, Longden KD, Nern A et al (2021) Synaptic targets of photoreceptors specialized to detect color and skylight polarization in Drosophila. Elife. 10.7554/eLife.71858 34913436 10.7554/eLife.71858 PMC8789284

[CR73] Kitamoto J, Sakamoto K, Ozaki K et al (1998) Two visual pigments in A single photoreceptor cell: identification and histological localization of three Mrnas encoding visual pigment Opsins in the retina of the butterfly Papilio Xuthus. J Exp Biol 201:1255–1261. 10.1242/jeb.201.9.1255 9547302 10.1242/jeb.201.9.1255

[CR74] Kolb G (1985) Ultrastructure and adaptation in the retina of Aglais urticae (Lepidoptera). Zoomorphology 105:90–98. 10.1007/BF00312143

[CR75] van der Kooi CJ, Stavenga DG, Arikawa K et al (2021) Evolution of insect color vision: from spectral sensitivity to visual ecology. Annu Rev Entomol 66:435–461. 10.1146/annurev-ento-061720-071644 32966103 10.1146/annurev-ento-061720-071644

[CR76] Koshitaka H, Kinoshita M, Vorobyev M, Arikawa K (2008) Tetrachromacy in a butterfly that has eight varieties of spectral receptors. Proc R Soc B Biol Sci 275:947–954. 10.1098/rspb.2007.1614 10.1098/rspb.2007.1614 PMC2599938 18230593

[CR77] Kumar JP (2012) Building an Ommatidium one cell at a time. Dev Dyn 241:136–149. 10.1002/dvdy.23707 22174084 10.1002/dvdy.23707 PMC3427658

[CR78] Labhart T, Meyer EP (1999) Detectors for polarized skylight in insects: a survey of ommatidial specializations in the dorsal rim area of the compound eye. Microsc Res Tech 47:368–379 10607378 10.1002/(SICI)1097-0029(19991215)47:6<368::AID-JEMT2>3.0.CO;2-Q

[CR79] Labhart T, Meyer EP (2002) Neural mechanisms in insect navigation: polarization compass and odometer. Curr Opin Neurobiol 12:707–714. 10.1016/S0959-4388(02)00384-7 12490263 10.1016/s0959-4388(02)00384-7

[CR80] Labhart T, Baumann F, Bernard GD (2009) Specialized ommatidia of the polarization-sensitive dorsal rim area in the eye of monarch butterflies have non-functional reflecting tapeta. Cell Tissue Res 338:391–400. 10.1007/s00441-009-0886-7 19876649 10.1007/s00441-009-0886-7 PMC2779342

[CR81] Land MF, Nilsson D-E (2012) Superposition eyes. Animal eyes. Oxford University Press, pp 191–214

[CR82] Langer H, Struwe G (1972) Spectral absorption by screening pigment granules in the compound eye of butterflies (Heliconius). J Comp Physiol 79:203–212. 10.1007/BF00697773

[CR83] Langer H, Hamann B, Meinecke CC (1979) Tetrachromatic visual system in the moth Spodoptera exempta (Insecta: Noctuidae). J Comp Physiol A 129:235–239. 10.1007/BF00657659

[CR84] Liénard MA, Bernard GD, Allen A et al (2021) The evolution of red color vision is linked to coordinated rhodopsin tuning in Lycaenid butterflies. Proc Natl Acad Sci 118:1–12. 10.1073/pnas.2008986118 10.1073/pnas.2008986118 PMC8017955 33547236

[CR85] Linzen B (1974) The tryptophan → ommochrome pathway in insects. In: Advances in insect physiology. Academic Press, pp 117–246

[CR86] Matsushita A, Awata H, Wakakuwa M et al (2012) Rhabdom evolution in butterflies: insights from the uniquely tiered and heterogeneous ommatidia of the glacial Apollo butterfly, Parnassius glacialis. Proc R Soc B Biol Sci 279:3482–3490. 10.1098/rspb.2012.0475 10.1098/rspb.2012.0475 PMC3396891 22628477

[CR87] Matsushita A, Stewart F, Ilić M et al (2022) Connectome of the lamina reveals the circuit for early color processing in the visual pathway of a butterfly. Curr Biol 32:2291–2299e3. 10.1016/j.cub.2022.03.066 35439432 10.1016/j.cub.2022.03.066

[CR88] Mazzoni EO, Desplan C, Çelik A (2004) One receptor’ rules in sensory neurons. Dev Neurosci 26:388–395. 10.1159/000082281 15855768 10.1159/000082281

[CR89] Mazzoni EO, Celik A, Wernet MF et al (2008) Iroquois complex genes induce Co-Expression of rhodopsins in Drosophila. PLoS Biol 6:e97. 10.1371/journal.pbio.0060097 18433293 10.1371/journal.pbio.0060097 PMC2323304

[CR90] McCulloch KJ, Osorio D, Briscoe AD (2016) Sexual dimorphism in the compound eye of Heliconius erato: a nymphalid butterfly with at least five spectral classes of photoreceptor. J Exp Biol 219:2377–2387. 10.1242/jeb.136523 27247318 10.1242/jeb.136523

[CR91] McCulloch KJ, Yuan F, Zhen Y et al (2017) Sexual dimorphism and retinal mosaic diversification following the evolution of a Violet receptor in butterflies. Mol Biol Evol 34:2271–2284. 10.1093/MOLBEV/MSX163 28505307 10.1093/molbev/msx163

[CR92] McCulloch KJ, Macias-Muñoz A, Briscoe AD (2022a) Insect opsins and evo-devo: what have we learned in 25 years? Philos Trans R Soc B Biol Sci. 10.1098/rstb.2021.0288 10.1098/rstb.2021.0288 PMC9441233 36058243

[CR93] McCulloch KJ, Macias-Muñoz A, Mortazavi A, Briscoe AD (2022b) Multiple mechanisms of photoreceptor spectral tuning in *Heliconius* butterflies. Mol Biol Evol. 10.1093/molbev/msac067 35348742 10.1093/molbev/msac067 PMC9048915

[CR94] Meinecke C (1981) The fine structure of the compound eye of the African armyworm moth, Spodoptera exempta walk. (Lepidoptera, Noctuidae). Cell Tissue Res 216:333–347. 10.1007/BF00233623 7226213 10.1007/BF00233623

[CR95] Meyer-Rochow VB, Gál J (2004) Dimensional limits for arthropod eyes with superposition optics. Vis Res 44:2213–2223. 10.1016/j.visres.2004.04.009 15208008 10.1016/j.visres.2004.04.009

[CR96] Meyer-Rochow VB, Lindström M (2025) Reflections of an insect’s lifestyle and habitat: morphological and ultrastructural adaptations involving the eyes of insects. Insect ecomorphology. Elsevier, pp 93–153

[CR97] Meyer-Rochow VB, Lau (Stanley) TF (2008) Sexual dimorphism in the compound eye of the moth Operophtera brumata (Lepidoptera, Geometridae. Invertebr Biol 127:201–216. 10.1111/j.1744-7410.2008.00131.x

[CR98] Miller WH, Bernard GD (1968) Butterfly glow. J Ultrastruct Res 24:286–294. 10.1016/S0022-5320(68)90065-8 5704880 10.1016/s0022-5320(68)90065-8

[CR99] Mitter C, Davis DR, Cummings MP (2017) Phylogeny and evolution of Lepidoptera. Annu Rev Entomol 62:265–283. 10.1146/annurev-ento-031616-035125 27860521 10.1146/annurev-ento-031616-035125

[CR100] Mohr T, Meinertzhagen IA, Fischer S (2020) Novel type of sub-retinal pigment shield in the miniaturized compound eye of Trichogramma evanescens. J Comp Neurol 528:167–174. 10.1002/cne.24745 31306484 10.1002/cne.24745

[CR101] Monsma SA, Booker R (1996) Genesis of the adult retina and outer optic lobes of the moth,manduca sexta. I. Patterns of proliferation and cell death. J Comp Neurol 367:10–20 8867280 10.1002/(SICI)1096-9861(19960325)367:1<10::AID-CNE2>3.0.CO;2-M

[CR102] Mulhair PO, Crowley L, Boyes DH et al (2023) Opsin gene duplication in lepidoptera: retrotransposition, sex linkage, and gene expression. Mol Biol Evol 40:1–14. 10.1093/molbev/msad241 10.1093/molbev/msad241 PMC10642689 37935057

[CR103] Nagloo N, Kinoshita M, Arikawa K (2020) Spectral organization of the compound eye of a migrating nymphalid, the Chestnut tiger butterfly, *Parantica sita*. J Exp Biol. 10.1242/jeb.217703 31900350 10.1242/jeb.217703

[CR104] Nilsson D-E (1989) Optics and evolution of the compound eye. Facets of vision. Springer Berlin Heidelberg, Berlin, Heidelberg, pp 30–73

[CR105] Ogawa Y, Awata H, Wakakuwa M et al (2012) Coexpression of three middle wavelength-absorbing visual pigments in sexually dimorphic photoreceptors of the butterfly Colias erate. J Comp Physiol A 198:857–867. 10.1007/s00359-012-0756-8 10.1007/s00359-012-0756-8 22972231

[CR106] Ogawa Y, Kinoshita M, Stavenga DG, Arikawa K (2013) Sex-specific retinal pigmentation results in sexually dimorphic long-wavelength-sensitive photoreceptors in the Eastern pale clouded yellow butterfly, Colias erate. J Exp Biol 216:1916–1923. 10.1242/jeb.083485 23393285 10.1242/jeb.083485

[CR107] Orridge GAH, Giddings C, Stange G (1972) The superposition eye of skipper butterflies. Proc R Soc Lond Ser B Biol Sci 182:457–495. 10.1098/rspb.1972.0088

[CR108] Osorio D (2007) Spam and the evolution of the fly’s eye. BioEssays 29:111–115. 10.1002/bies.20533 17226795 10.1002/bies.20533

[CR109] Perry M, Kinoshita M, Saldi G et al (2016) Molecular logic behind the three-way stochastic choices that expand butterfly colour vision. Nature 535:280–284. 10.1038/nature18616 27383790 10.1038/nature18616 PMC4988338

[CR110] Pirih P, Arikawa K, Stavenga DG (2010) An expanded set of photoreceptors in the Eastern pale clouded yellow butterfly, Colias erate. J Comp Physiol A 196:501–517. 10.1007/s00359-010-0538-0 10.1007/s00359-010-0538-0 PMC2890080 20524001

[CR111] Pirih P, Ilić M, Rudolf J et al (2018) The giant butterfly-moth Paysandisia archon has spectrally rich apposition eyes with unique light-dependent photoreceptor dynamics. J Comp Physiol A 204:639–651. 10.1007/s00359-018-1267-z 10.1007/s00359-018-1267-z PMC6028894 29869100

[CR112] Pirih P, Meglič A, Stavenga D et al (2020) The red Admiral butterfly’s living light sensors and signals. Faraday Discuss 223:81–97. 10.1039/D0FD00075B 32760932 10.1039/d0fd00075b

[CR113] Pirih P, Ilić M, Meglič A, Belušič G (2022) Opponent processing in the retinal mosaic of nymphalid butterflies. Philos Trans R Soc B Biol Sci 377:20210275. 10.1098/rstb.2021.0275 10.1098/rstb.2021.0275 PMC9441239 36058238

[CR114] Qiu X, Arikawa K (2003) Polymorphism of red receptors: sensitivity spectra of proximal photoreceptors in the small white butterfly Pieris rapae crucivora. J Exp Biol 206:2787–2793. 10.1242/jeb.00493 12847124 10.1242/jeb.00493

[CR115] Qiu X, Vanhoutte K, Stavenga D, Arikawa K (2002) Ommatidial heterogeneity in the compound eye of the male small white butterfly, Pieris rapae crucivora. Cell Tissue Res 307:371–379. 10.1007/s00441-002-0517-z 11904774 10.1007/s00441-002-0517-z

[CR116] Reinke R, Zipursky SL (1988) Cell-cell interaction in the drosophila retina: the bride of sevenless gene is required in photoreceptor cell R8 for R7 cell development. Cell 55:321–330. 10.1016/0092-8674(88)90055-4 3167983 10.1016/0092-8674(88)90055-4

[CR117] Reppert SM, Zhu H, White RH (2004) Polarized light helps monarch butterflies navigate. Curr Biol 14:155–158. 10.1016/j.cub.2003.12.034 14738739 10.1016/j.cub.2003.12.034

[CR118] Ribi W (1978) Ultrastructure and migration of screening pigments in the retina of Pieris rapae L. (Lepidoptera, Pieridae). Cell Tissue Res 191:57–73. 10.1007/BF00223215 688357 10.1007/BF00223215

[CR119] Ribi WA (1979) Structural differences in the tracheal tapetum of diurnal butterflies. Z Für Naturforsch C 34:284–287. 10.1515/znc-1979-3-421

[CR120] Ribi W (1987) Anatomical identification of spectral receptor types in the retina and lamina of the Australian orchard butterfly, Papilio Aegeus Aegeus D. Cell Tissue Res 247:393–407. 10.1007/BF00218321

[CR121] Rossi M, Hausmann AE, Alcami P et al (2024) Adaptive introgression of a visual preference gene. Sci (80-) 383:1368–1373. 10.1126/science.adj9201 10.1126/science.adj9201 PMC7616200 38513020

[CR122] Saito T, Koyanagi M, Sugihara T et al (2019) Spectral tuning mediated by helix III in butterfly long wavelength-sensitive visual Opsins revealed by heterologous action spectroscopy. Zool Lett 5:35. 10.1186/s40851-019-0150-2 10.1186/s40851-019-0150-2 PMC6915953 31890273

[CR123] Satoh A, Kinoshita M, Arikawa K (2016) Innate preference and learning of colour in the male cotton bollworm Helicoverpa armigera. J Exp Biol 219:3857–3860. 10.1242/jeb.148064 27802146 10.1242/jeb.148064

[CR124] Satoh A, Stewart FJ, Koshitaka H et al (2017) Red-shift of spectral sensitivity due to screening pigment migration in the eyes of a moth, Adoxophyes Orana. Zool Lett 3:14. 10.1186/s40851-017-0075-6 10.1186/s40851-017-0075-6 PMC5575869 28861276

[CR125] Sauman I, Briscoe AD, Zhu H et al (2005) Connecting the navigational clock to sun compass input in monarch butterfly brain. Neuron 46:457–467. 10.1016/j.neuron.2005.03.014 15882645 10.1016/j.neuron.2005.03.014

[CR126] Schnaitmann C, Haikala V, Abraham E et al (2018) Color processing in the early visual system of Drosophila. Cell 172:318–330e18. 10.1016/j.cell.2017.12.018 29328919 10.1016/j.cell.2017.12.018

[CR127] Schnaitmann C, Pagni M, Reiff DF (2020) Color vision in insects: insights from Drosophila. J Comp Physiol 2020 2062 206:183–198. 10.1007/S00359-019-01397-3 10.1007/s00359-019-01397-3 PMC7069916 32020291

[CR128] Buerkle NP, VanKuren NW, Westerman EL et al (2022) Sex-limited diversification of the eye in *Heliconius* butterflies. bioRxiv 2022.04.25.489414 10.1007/s00359-025-01768-z PMC12978942 41102534

[CR129] Shimizu I, Yamakawa Y, Shimazaki Y, Iwasa T (2001) Molecular cloning of Bombyx cerebral Opsin (Boceropsin) and cellular localization of its expression in the silkworm brain. Biochem Biophys Res Commun 287:27–34. 10.1006/bbrc.2001.5540 11549248 10.1006/bbrc.2001.5540

[CR130] Shimohigashi M, Tominaga Y (1986) The compound eye of Parnara guttata (Insecta, lepidoptera, Hesperiidae): Fine structure of the ommatidium. Zoomorphology 106:131–136. 10.1007/BF00312201

[CR131] Shimohigashi M, Tominaga Y (1991) Identificaton of UV, green and red receptors, and their projection to lamina in the cabbage butterfly, Pieris rapae. Cell Tissue Res 263:49–59. 10.1007/BF00318399

[CR132] Shimohigashi M, Tominaga Y (1999) Synaptic organization in the lamina of the superposition eye of a skipper butterfly, Parnara guttata. J Comp Neurol 408:107–124 10331583 10.1002/(sici)1096-9861(19990524)408:1<107::aid-cne8>3.0.co;2-#

[CR133] Sison-Mangus MP, Bernard GD, Lampel J, Briscoe AD (2006) Beauty in the eye of the beholder: the two blue Opsins of Lycaenid butterflies and the Opsin gene-driven evolution of sexually dimorphic eyes. J Exp Biol 209:3079–3090. 10.1242/jeb.02360 16888057 10.1242/jeb.02360

[CR134] Sison-Mangus MP, Briscoe AD, Zaccardi G et al (2008) The Lycaenid butterfly polyommatus Icarus uses a duplicated blue Opsin to see green. J Exp Biol 211:361–369. 10.1242/jeb.012617 18203991 10.1242/jeb.012617

[CR135] Sondhi Y, Ellis EA, Bybee SM et al (2021) Light environment drives evolution of color vision genes in butterflies and moths. Commun Biol 4:177. 10.1038/s42003-021-01688-z 33564115 10.1038/s42003-021-01688-z PMC7873203

[CR136] Song B-M, Lee C-H (2018) Toward a mechanistic Understanding of color vision in insects. Front Neural Circuits 12:1–9. 10.3389/fncir.2018.00016 29527156 10.3389/fncir.2018.00016 PMC5829095

[CR137] Stalleicken J, Mukhida M, Labhart T et al (2005) Do monarch butterflies use polarized skylight for migratory orientation? J Exp Biol 208:2399–2408. 10.1242/jeb.01613 15939779 10.1242/jeb.01613

[CR138] Stavenga DG (1995) Insect retinal pigments: spectral characteristics and physiological functions. Prog Retin Eye Res 15:231–259. 10.1016/1350-9462(95)00011-9

[CR139] Stavenga DG (2002) Reflections on colourful ommatidia of butterfly eyes. J Exp Biol 205:1077–1085. 10.1242/jeb.205.8.1077 11919267 10.1242/jeb.205.8.1077

[CR140] Stavenga DG, Arikawa K (2006) Evolution of color and vision of butterflies. Arthropod Struct Dev 35:307–318. 10.1016/j.asd.2006.08.011 18089078 10.1016/j.asd.2006.08.011

[CR141] Stavenga DG, Kuiper JW (1977) Insect pupil mechanisms. J Comp Physiol A 113:55–72. 10.1007/BF00610453

[CR142] Stewart FJ, Kinoshita M, Arikawa K (2015) The butterfly Papilio xuthus detects visual motion using chromatic contrast. Biol Lett. 10.1098/rsbl.2015.0687. 11:26490417 10.1098/rsbl.2015.0687 PMC4650181

[CR143] Takemura S, Stavenga DG, Arikawa K (2007) Absence of eye shine and tapetum in the heterogeneous eye of Anthocharis butterflies (Pieridae). J Exp Biol 210:3075–3081. 10.1242/jeb.002725 17704082 10.1242/jeb.002725

[CR144] Tang Y-H, Bi S-Y, Wang X-D et al (2024) Opsin mutants alter host plant selection by color vision in the nocturnal invasive pest Tuta absoluta. Int J Biol Macromol 265:130636. 10.1016/j.ijbiomac.2024.130636 38467214 10.1016/j.ijbiomac.2024.130636

[CR145] Thanawala SU, Rister J, Goldberg GW et al (2013) Regional modulation of a stochastically expressed factor determines photoreceptor subtypes in the Drosophila retina. Dev Cell 25:93–105. 10.1016/j.devcel.2013.02.016 23597484 10.1016/j.devcel.2013.02.016 PMC3660048

[CR146] Tomlinson A (2012) The origin of the Drosophila subretinal pigment layer. J Comp Neurol 520:2676–2682. 10.1002/cne.23063 22684937 10.1002/cne.23063 PMC3955728

[CR147] Uchiyama H, Awata H, Kinoshita M, Arikawa K (2013) Rough eyes of the Northeast-Asian wood white Leptidea amurensis. J Exp Biol 216:3414–3421. 10.1242/jeb.089169 23685978 10.1242/jeb.089169

[CR148] VanKuren NW, Buerkle NP, Lu W et al (2025) Genetic, developmental, and neural changes underlying the evolution of butterfly mate preference. PLOS Biol 23:e3002989. 10.1371/journal.pbio.3002989 40067994 10.1371/journal.pbio.3002989 PMC12136153

[CR149] Wahlberg N, Wheat CW, Peña C (2013) Timing and patterns in the taxonomic diversification of Lepidoptera (Butterflies and Moths). PLoS ONE 8:e80875. 10.1371/journal.pone.0080875 24282557 10.1371/journal.pone.0080875 PMC3839996

[CR150] Wainwright JB, Schofield C, Conway M et al (2023) Multiple axes of visual system diversity in Ithomiini, an ecologically diverse tribe of mimetic butterflies. J Exp Biol. 10.1242/jeb.246423 37921078 10.1242/jeb.246423 PMC10714147

[CR151] Wakakuwa M, Kurasawa M, Giurfa M, Arikawa K (2005) Spectral heterogeneity of honeybee ommatidia. Naturwissenschaften 92:464–467. 10.1007/s00114-005-0018-5 16136295 10.1007/s00114-005-0018-5

[CR152] Wakakuwa M, Terakita A, Koyanagi M et al (2010) Evolution and mechanism of spectral tuning of Blue-Absorbing visual pigments in butterflies. PLoS ONE 5:e15015. 10.1371/journal.pone.0015015 21124838 10.1371/journal.pone.0015015 PMC2991335

[CR153] Warrant EJ, McIntyre PD (1996) The visual ecology of pupillary action in superposition eyes. J Comp Physiol A 178:75–90. 10.1007/BF00189592

[CR154] Warrant E, Somanathan H (2022) Colour vision in nocturnal insects. Philos Trans R Soc B Biol Sci. 10.1098/rstb.2021.0285 10.1098/rstb.2021.0285 PMC9441242 36058247

[CR155] Wernet MF, Mazzoni EO, Çelik A et al (2006) Stochastic spineless expression creates the retinal mosaic for colour vision. Nature 440:174–180. 10.1038/nature04615 16525464 10.1038/nature04615 PMC3826883

[CR156] Wernet MF, Velez MM, Clark DA et al (2012) Genetic dissection reveals two separate retinal substrates for polarization vision in Drosophila. Curr Biol 22:12–20. 10.1016/j.cub.2011.11.028 22177904 10.1016/j.cub.2011.11.028 PMC3258365

[CR157] Wernet MF, Perry MW, Desplan C (2015) The evolutionary diversity of insect retinal mosaics: common design principles and emerging molecular logic. Trends Genet 31:316–328. 10.1016/j.tig.2015.04.006 26025917 10.1016/j.tig.2015.04.006 PMC4458154

[CR158] White RH, Xu H, Münch TA et al (2003) The retina of Manduca sexta: rhodopsin expression, the mosaic of green-, blue- and UV-sensitive photoreceptors, and regional specialization. J Exp Biol 206:3337–3348. 10.1242/jeb.00571 12939366 10.1242/jeb.00571

[CR159] Wiens JJ, Lapoint RT, Whiteman NK (2015) Herbivory increases diversification across insect clades. Nat Commun 6:8370. 10.1038/ncomms9370 26399434 10.1038/ncomms9370 PMC4598556

[CR160] Wright CJ, Stevens L, Mackintosh A et al (2024a) Comparative genomics reveals the dynamics of chromosome evolution in Lepidoptera. Nat Ecol Evol 8:777–790. 10.1038/s41559-024-02329-4 38383850 10.1038/s41559-024-02329-4 PMC11009112

[CR161] Wright DS, Rodriguez-Fuentes J, Ammer L et al (2024b) Selection drives divergence of eye morphology in sympatric Heliconius butterflies. Evol (N Y) 78:1338–1346. 10.1093/evolut/qpae073 10.1093/evolut/qpae073 PMC7616201 38736286

[CR162] Xia Q, Zhou Z, Lu C et al (2004) A draft sequence for the genome of the domesticated silkworm (Bombyx mori). Sci (80-) 306:1937–1940. 10.1126/science.1102210 10.1126/science.1102210 15591204

[CR163] Yack JE, Johnson SE, Brown SG, Warrant EJ (2007) The eyes of macrosoma sp. (Lepidoptera: Hedyloidea): A nocturnal butterfly with superposition optics. Arthropod Struct Dev 36:11–22. 10.1016/j.asd.2006.07.001 18089084 10.1016/j.asd.2006.07.001

[CR164] Yang X, Ran H, Jiang Y et al (2024) Fine structure of the compound eyes of the crepuscular moth grapholita molesta (Busck 1916) (Lepidoptera: Tortricidae). Front Physiol 15:1343702. 10.3389/fphys.2024.1343702 38390450 10.3389/fphys.2024.1343702 PMC10883378

[CR165] Zaccardi G, Kelber A, Sison-Mangus MP, Briscoe AD (2006) Color discrimination in the red range with only one long-wavelength sensitive Opsin. J Exp Biol 209:1944–1955. 10.1242/jeb.02207 16651559 10.1242/jeb.02207

[CR166] Zhan S, Merlin C, Boore JL, Reppert SM (2011) The monarch butterfly genome yields insights into Long-Distance migration. Cell 147:1171–1185. 10.1016/j.cell.2011.09.052 22118469 10.1016/j.cell.2011.09.052 PMC3225893

