# Correction: Cellular innovations and diversity in the lepidopteran compound eye

**DOI:** 10.1007/s00359-025-01758-1

**Published:** 2025-11-13

**Authors:** Wei Lu, Marcus R. Kronforst

**Affiliations:** Department of Ecology and Evolution, University of Chicago, Chicago, IL 60637 USA

**Correction: Journal of Comparative Physiology A**

10.1007/s00359-025-01751-8

In the corrected version of the article, the legend of Fig. 1 was moved below the figure. The legend of Fig. 2, which was missing in the original article, was added below the figure in the corrected version of the article.

Incorrect Version

**Fig. 2:**
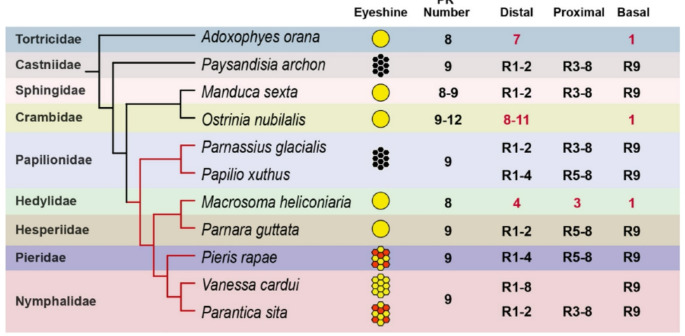
XXX

Corrected Version

**Fig. 2:**
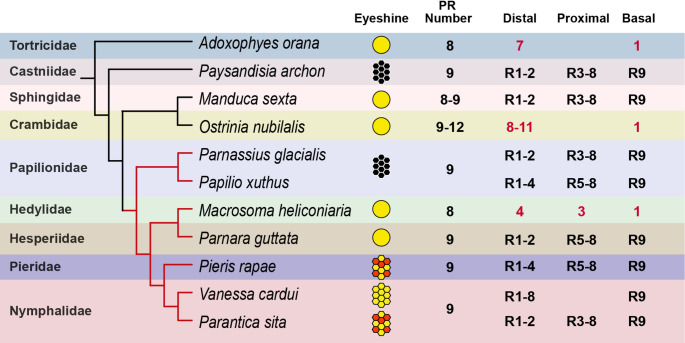
Evolution of ommatidial anatomical structures in Lepidoptera. A phylogeny of representative species from various Lepidoptera families is shown, with butterflies (Superfamily Papilionoidea) highlighted in red branches. The family-level phylogeny is based on (Kawahara et al. 2019). In the eyeshine column: colorful hexagons, apposition eyes with heterogeneous eyeshine; yellow hexagons, apposition eyes with homogeneous eyeshine; black hexagons, apposition eyes without eyeshine; yellow circles, superposition eyes with eyeshine. For each species, the total number of photoreceptors per ommatidium is indicated, along with their grouping based on their contribution to the rhabdom, which is organized into two or three tiers. Photoreceptor naming follows the Ribi (1978) scheme. For species where photoreceptor homologies are uncertain, the number of photoreceptors in each tier is indicated (in red text). Across all species, regardless of eye type (apposition or superposition), the ommatidium consistently contains a distinct basal photoreceptor. References: *Adoxophyes* (Satoh et al. 2017); *Paysandisia* (Pirih et al. 2018); *Manduca* (White et al. 2003; Gao et al. 2025); *Ostrinia* (Belušič et al. 2017); *Parnassius* (Matsushita et al. 2012); *Papilio* (Arikawa and Uchiyama 1996); *Macrosoma* (Yack et al. 2007); *Parnara* (Shimohigashi and Tominaga 1986); *Pieris *(Ribi 1978); *Vanessa* (Briscoe et al. 2003); *Parantica* (Nagloo et al. 2020).

The original article has been corrected.

